# Shu Gan Jie Yu herbal combination enhances tamoxifen sensitivity in breast cancer cells via modulation of the SNCG/*p*-ERα,mTOR /AKT and *p*-ERK pathway

**DOI:** 10.3389/fphar.2026.1868856

**Published:** 2026-07-27

**Authors:** Chu Chen, Shuo Sun, Weide Zhang, Xinhui Zhang, Wenjie Zhang, Yi Zhao, Youzhi Sun

**Affiliations:** 1 School of Chinese Medicine, Jiangxi University of Chinese Medicine, Nanchang, China; 2 Research Center for Differentiation and Development of Basic Theory of Traditional Chinese Medicine, Jiangxi University of Chinese Medicine, Nanchang, China

**Keywords:** estrogen receptor-positive breast cancer, mTOR /AKT, p-ERK pathway, Shu Gan Jie Yu, SNCG/p-ERα, synergy, tamoxifen

## Abstract

**Objective:**

This study aims to investigate the therapeutic effects and potential mechanisms of *Shu Gan Jie Yu herbal combination* (*Cyperus rotundus* L.*, Citrus medica* L*.* var. *Sarcodactylis Swingle and Rosa rugosa Thunb*; CCR) combined with Tamoxifen (TAM) in inhibiting estrogen receptor-positive (ER+) breast cancer.

**Methods:**

The water extract of the CCR was tested on MCF-7, T47D cell lines, and their SNCG-overexpressing counterparts. The CCK8 assay was used to evaluate the effects of CCR, TAM alone, and their combined application. CompuSyn and SynergyFinder software were employed to assess whether the combination therapy exhibited synergistic effects. Colony formation assays and flow cytometry were conducted to examine the impact on proliferation and apoptosis of SNCG-overexpressing cells, respectively. Co-immunoprecipitation (CoIP) and dual-luciferase reporter gene assays were used to investigate the interaction between SNCG and ERα. Western blot analysis was performed to evaluate the effects of CCR and TAM, alone or in combination, on the expression of ERα, SNCG, and phosphorylated proteins in MCF-7 and T47D cells, as well as on the expression of ERα, SNCG, EGFR, AKT, mTOR, MAPK, ERK and their phosphorylated forms in SNCG-overexpressing cells.

**Results:**

In our study, treatment with CCR enhanced the inhibitory effects of TAM on the breast cancer cell lines examined. CoIP and dual-luciferase reporter gene assays indicated a potential protein–protein interaction between SNCG and ERα, suggesting that SNCG overexpression enhanced ERα promoter activity. Overexpression of SNCG led to the upregulation of ERα. CCR combined with TAM markedly suppressed cell migration and colony formation, and significantly induced apoptosis. These effects were mechanistically associated with reduced protein expression levels of SNCG, *p*-ERα, and key molecules in the mTOR/AKT and *p*-ERK signaling pathways.

**Conclusion:**

Our results indicate that CCR exhibits potent anti-proliferative activity against ER + breast cancer. Moreover, the combination of CCR and TAM synergistically enhances the anti-proliferative activity, at least in part via modulation of the SNCG/*p*-ERα, inhibiting the mTOR/AKT and *p*-ERK signaling pathways.

## 1 Introduction

Breast cancer is the leading threat to women’s health globally (Kim et al., 2025). Currently, ER+ and/or progesterone receptor-positive (PR+) breast cancers account for approximately 75% of all breast cancer cases. Due to their hormone signal-dependent growth characteristics, they have become a primary focus in clinical prevention and treatment (Roßwag et al., 2021). Continuous research on breast cancer prevention and treatment has always been a hot and challenging topic. Endocrine therapy is one of the effective treatment methods for this type of breast cancer. TAM has been established as the “gold standard” drug (Javankiani et al., 2025; Schroth et al., 2025),its mechanism of action lies in inhibiting tumor cell proliferation by competitively binding to estrogen receptors. However, long-term Endocrine therapy faces two major challenges: 30%–40% treatment failure due to primary or secondary resistance (Lei et al., 2019) and the induction of adverse reactions such as the increased incidence of endometrial cancer, significantly affecting patients’ quality of life (Ghanavati et al., 2023; Ryu et al., 2022). In recent years, several studies have reported that combined treatment with traditional Chinese medicine can significantly enhance the therapeutic sensitivity of TAM and reduce its side effects (Chen et al., 2021; Lu et al., 2024; Zhao W. et al., 2024). For example, triptolide, a pentacyclic terpene compound derived from the root of the traditional Chinese medicinal plant Tripterygium wilfordii, has effects such as promoting apoptosis and anti-cancer properties (Kashyap et al., 2018), study find that combined with TAM exerts synergistic anticancer effects by inducing apoptosis and autophagy in MCF-7 cells (Wang et al., 2021). Trametes robiniophila murris is a sandy beige mushroom and a traditional Chinese medicine with a history of 1,600 years (Sun et al., 2013; Chen et al., 2018), it has anti-tumor properties (Li et al., 2020a), regulates immune function (Long and Wu, 2023). Research has shown that its extract works synergistically with tamoxifen to inhibit the AKT signaling pathway, inducing autophagy and apoptosis in ER + breast cancer cells (Qi et al., 2016). Zligustilide is the main active component of the traditional Chinese medicine *Angelica sinensis*. It is a phthalide compound and is also the source of the aromatic scent of the root of *A. sinensis* (Wedge et al., 2009; Zeng et al., 2015), it has been shown to enhance the sensitivity of TAM to ERα breast cancer cells MDA-MB-231 (Ma et al., 2017). This finding suggests a broad clinical application prospect for combination drug therapy in breast cancer.

According to traditional Chinese medicine (TCM), breast cancer is closely associated with emotional disturbance and subsequent liver qi stagnation (Wu et al., 2025a; Qiu et al., 2022). Guided by TCM therapeutic principles, we formulated the herbal combination based on syndrome differentiation, which is named “Jiao Yao” (Song et al., 2017). Guided by the liver-soothing and depression-relieving principle of traditional Chinese medicine, we developed CCR, a compound consisting of three medicinal herbs. Our previous study demonstrated that CCR exerts anti-breast cancer effects by regulating the SNCG/ER-α/AKT-ERK pathway (Zhao Y. et al., 2024). However, whether CCR can potentiate the therapeutic efficacy of TAM remains unexplored. Given their complementary mechanisms—TAM as a selective estrogen receptor modulator and CCR as a multi-targeting agent modulating the SNCG/ER-α/AKT-ERK axis—we hypothesized that CCR may compensate for the inherent limitations of TAM monotherapy and achieve superior anti-tumor activity through synergistic combination. Synucleins include α-synuclein (SNCA), β-synuclein (SNCB), and γ-synuclein (SNCG), which are involved in neurodegenerative diseases and cancer (Venati and Uversky, 2024), SNCG is not only highly expressed in ER + breast cancer, but also closely associated with tumor proliferation, invasion, and endocrine resistance (Zanotti et al., 2023). Additionally, it can regulate ERα activity by incorporating into the heat shock protein (Hsp)-based chaperone complex (Jiang et al., 2004). The PI3K/AKT/mTOR signaling pathway is aberrantly hyperactivated in ER + breast cancer (Hernandez-Aya and Gonzalez-Angulo, 2011; Alves and Ditzel, 2023), emerging evidence has indicated that crosstalk between ERα and the PI3K/AKT/mTOR pathway accelerates the progression of ER + breast cancer (Cuesta et al., 2019).

Combination therapy is based on the positive interactions (synergistic or additive effects) between two or more drugs, with synergistic interactions yielding more potent therapeutic outcomes. Combination therapy typically uses lower doses of two compounds acting on distinct molecular pathways, thus showing lower toxicity than monotherapy. It also confers multiple benefits, such as reduced drug concentration and toxicity, enhanced therapeutic efficacy, targeted modulation of multiple molecular pathways, and improved cellular treatment sensitivity (Milczarek et al., 2021; Chen et al., 2020). Therefore, in the present study, our research aims to evaluate whether CCR can enhance the antitumor effects of TAM and to elucidate its underlying antitumor mechanisms.

## 2 Materials and methods

### 2.1 Cell lines

MCF-7 (SCSP-531), T47D (SCSP-564), and MCF-10A (GNHu50) were all provided by the Cell Bank of the Chinese Academy of Sciences, they were respectively cultured in high-glucose DMEM and RPMI 1640 (Gibco, New York, United States of America).

### 2.2 Preparation of CCR

CCR contains *Cyperus rotundus* L., *Citrus medica* L. var. *Sarcodactylis Swingle* and *Rosa rugosa Thunb*, each weighed at 250 g according to a 1:1:1 ratio. Soak the herbs in 10 times their weight of pure water for 30 min. Heat and reflux extract for 2 h. Collect the filtrate. Then add 8 times the weight of water to the residue for a second extraction, extract for 1 h, and filter. Combine the two extracts and concentrate the combined liquid under reduced pressure using a rotary evaporator. Further process the concentrate into a freeze-dried powder. All medicinal materials were purchased from JiangzhongTraditional Chinese Medicine Pieces Company (Nanchang, China).

### 2.3 High-performance liquid chromatography and mass spectrometry

HPLC analysis was performed using a Waters ACQUITY UPLC HSS T3 column (2.1 mm × 100 mm, 1.8 μm), paired with a Waters ACQUITY UPLC HSS T3 guard column (2.1 mm × 5 mm, 1.8 μm). The column temperature is set at 40 °C, and the sample chamber temperature is maintained at 10 °C. The mobile phase consists of 0.1% formic acid in water and acetonitrile, with a flow rate of 0.3 mL/min. The injection volume is 3 μL.

Mass Spectrometry Conditions: A SYNAPT G2-Si Q-TOF/MS high-resolution ion mobility mass spectrometer is used, operating in MSE scanning mode. The ESI source temperature is set at 100 °C, with a cone gas flow rate of 50 L/h. The desolvation gas temperature is 400 °C, and its flow rate is maintained at 800 L/h. In positive ion mode, the capillary voltage is set to 3 Kv. In negative ion mode, it is set to 2.5 kV. The cone voltage is uniformly set at 40 V for both modes. LC-MS data were processed using UNIFI 1.9 software, and compounds were identified by database matching (mass deviation < ±10 ppm).

### 2.4 SNCG overexpression

Stable SNCG overexpression cell lines were established in MCF-7 and T47D cells via lentiviral infection. The SNCG coding sequence was inserted into pcDNA3.1 (+), and the overexpression lentivirus LV-SNCG was provided by GeneChem. The optimal multiplicity of infection was determined, and infection conditions were optimized using HiTransG A/P transduction enhancer. The minimum lethal concentration of puromycin was identified for subsequent positive cell selection. When infection efficiency reached approximately 80%, stable clones were selected and expanded in medium containing puromycin. Finally, the overexpression efficiency of SNCG in the established stable cell lines was validated by Real-time PCR.

### 2.5 Cell viability assay

Cell proliferation was assessed using the Cell Counting Kit-8 (APExBIO Technology LLC). T47D, MCF-7, MCF-10A, LV-SNCG-OE MCF-7, and LV-SNCG-OE T47D cells were seeded in 96-well plates at a density of 5 × 10 cells/mL. To evaluate the dose-dependent effects of single drugs, cells were treated with serially diluted CCR, TAM, or Ful. The CCR concentration gradient (0.078125–5 mg/mL, 2-fold serial dilution) was determined based on our previous study (Zhao W. et al., 2024), covering both sub-effective and effective doses for breast cancer cells. The TAM concentration range of 3–24 μM was selected according to published evidence, which fully spans the cytostatic and cytotoxic effects of TAM on ER + breast cancer cells (Wang et al., 2001; Wang, 2014; Reddel et al., 1985). The Ful concentration gradient of 2–18 μM was adopted based on common preclinical dosages for ER + breast cancer, covering the effective range for suppressing cell proliferation. For subsequent verification experiments, MCF-7, T47D, and MCF-10A cells were treated with optimized concentrations of TAM (7.5 μM and 11.25 μM), CCR (0.15625–1.25 mg/mL), and Ful (12 μM). After 24 h and 48 h of treatment, the optical density at 450 nm was detected using a multifunctional microplate reader (Tecan) to evaluate cell viability.

### 2.6 CompuSyn software and SynergyFinder investigate the inhibitory effect of combined CCR and TAM treatment on breast cancer

Using CompuSyn software, based on the Chou-Talalay Confidence Interval (CI) model, the interactions between drugs are quantitatively analyzed. A CI value less than 1 indicates a synergistic effect, meaning the combined drug effect is greater than the arithmetic sum of individual effects; a CI value equal to 1 indicates an additive effect; and a CI value greater than 1 indicates an antagonistic effect (Zhang et al., 2016). Additionally, the SynergyFinder tool (Ianevski et al., 2022)was used to calculate the Synergy Score, which is derived from dose-effect surface analysis of multiple concentration combinations to quantify the strength of synergy. A score greater than 0 indicates synergy, with higher values representing stronger synergy; a score equal to 0 indicates an additive effect; and a score less than 0 indicates antagonism.

### 2.7 Colony formation assay

Seed 2,500 LV-SNCG-OE T47D cells per well into six-well plates. Place the plates in a constant temperature incubator for culture. Once cell growth stabilizes, proceed with treatments as follows: control group, treatment groups with TAM at 11.25 μM, CCR at 0.625 mg/mL, combination treatment group, and positive drug control group with Ful at 12 μM. Treat for 12–16 days. After treatment, wash wells with PBS, then add 1 mL of 0.1% crystal violet staining solution per well and stain for 20 min. After staining, rinse several times with distilled water and air dry at room temperature.

### 2.8 Apoptosis assay

Transfected cells were treated with different drugs for 24 h, including TAM (11.25 μM), CCR (0.625 mg/mL), a combination of both drugs, and Ful (12 μM) as a positive control group. After treatment, cells were collected and stained with Annexin V-FITC and propidium iodide (PI) following standard protocols. The level of apoptosis was measured by flow cytometry (Cytoflex, Beckman), apoptosis rates were then quantified by flow cytometry analysis.

### 2.9 Cell scratch assay

First, use a marker pen to draw reference lines on the back of a 24-well plate. Then seed LV-SNCG-OE MCF-7 cells (2 × 10^5^ cells/well) and LV-SNCG-OE T47D cells (1 × 10^5^ cells/well) into the 24-well plate. After gently shaking the plate in a cross pattern to evenly distribute the cells, place it in a constant temperature incubator for culture. When the cells reach 100% confluence, use a sterile 200 μL pipette tip held vertically to the reference line to create a scratch. Next, wash the wells three times with PBS to remove the detached cells. Treatments are applied as follows: control group, TAM at 11.25 μM, CCR at 0.625 mg/mL, combination treatment group, and positive control group with Ful at 12 μM. Images of the scratch area are taken under a microscope at 0 h, 6 h, 12 h, and 24 h after incubation. Finally, the scratch healing rate is quantified using ImageJ software.

### 2.10 Western blot

T47D, MCF-7, LV-SNCG-OE MCF-7, and LV-SNCG-OE T47D cells at a density of 5 × 10^6^ cells per dish were evenly seeded into 100 mm culture dishes. The cells were divided into a control group, drug treatment groups with TAM at 11.25 μM, CCR at 0.46875 mg/mL or 0.625 mg/mL, a combination treatment group, and a positive drug control group with Ful at 12 μM. After treating the cells in the dishes for 24 h, the cells were collected and lysed with a protease inhibitor-containing lysis buffer to extract proteins. The protein samples were denatured by heating in a 100 °C water bath for 5 min. Protein concentration was determined using the BCA assay.

The protein samples were separated by SDS-PAGE and transferred onto PVDF membranes. After blocking, the membranes were incubated overnight at 4 °C with primary antibodies. Following washing with TBST, membranes were incubated with secondary antibodies at 37 °C for 2 h. After washing, an appropriate amount of developing solution was added, and the membranes were exposed and imaged using a chemiluminescence gel imaging system (ChemiDocXRS+,Bio-Rad Laboratories). Finally, the grayscale values of the target bands were analyzed using Image Lab 6.0 software.

### 2.11 Real-time PCR

Total RNA was extracted from the cells to be tested using the M5 Universal RNA Mini Kit (Mei5bio). cDNA synthesis was performed on a gradient PCR instrument (T100, Bio-Rad Laboratories) using the PrimeScript™ RT reagent Kit with gDNA Eraser (TaKaRa). Quantitative PCR was conducted using a high-throughput real-time fluorescence quantitative PCR system (CFX Connect, Bio-Rad Laboratories). The primer sequences for SNCG were forward 5′-GGAGGACTTGAGCCCATCTG and reverse 5′-CTCCTCTGC​CACTTCCTCTTTC (Sangon Biotech). Samples were prepared in triplicate, with β-actin used as the internal reference gene. Each reaction was repeated three times, and quantification was performed using the 2^-ΔΔCT^ method.

### 2.12 CoIP

Seed LV-SNCG-OE MCF-7 and LV-SNCG-OE T47D cells at a density of 2.5 × 10^6^ cells per 60 mm culture dish. Add 200 μL of lysis buffer containing inhibitors per dish, and lyse thoroughly. After centrifugation at 15,000 rpm for 15 min, collect the supernatant for CoIP. Incubate the target antibody or isotype control IgG with the supernatant at room temperature for 2 h to form antigen-antibody complexes. Then, mix with pre-treated magnetic bead suspension and incubate at room temperature for 1 h on a rotary mixer. Perform magnetic separation afterward. For every 20 μL of original magnetic bead volume, add 100 μL of Acid Elution Buffer, mix well, and incubate at room temperature on a rotary mixer for 5 min. After incubation, place the tube on a magnetic rack for 10 s to separate the beads, transfer the supernatant to a new centrifuge tube, and immediately add 10 μL of Neutralization Buffer. Mix appropriately.

### 2.13 Luciferase reporter gene assay

The ESR1 gene was cloned into the GV712 vector to construct the ESR1 expression plasmid, and the SNCG plasmid was constructed in the GV534 vector. Both plasmids were co-transfected into MCF-7 and T47D cells. After 24 h of transfection, the supernatant was collected, and luciferase activity was measured according to the kit instructions (Beyotime). Renilla luciferase was used as an internal control for normalization.

Relative Luciferase Expression = Firefly Luciferase RLU (measured from firefly luciferase assay)/Renilla Luciferase RLU (measured from Renilla luciferase assay).

### 2.14 Statistical analysis

Statistical analysis was performed using SPSS 25.0 and GraphPad Prism 8.0 software. Data are presented as mean ± standard deviation. The one-way ANOVA was used for comparisons among multiple groups, and independent samples t-test was used for comparisons between two groups. A *p*-value of less than 0.05 was considered statistically significant.

## 3 Results

### 3.1 Ultra-performance liquid chromatography coupled with quadrupole time-of-flight mass spectrometry (UPLC-Q-TOF-MS)analysis of CCR

**FIGURE 1:**
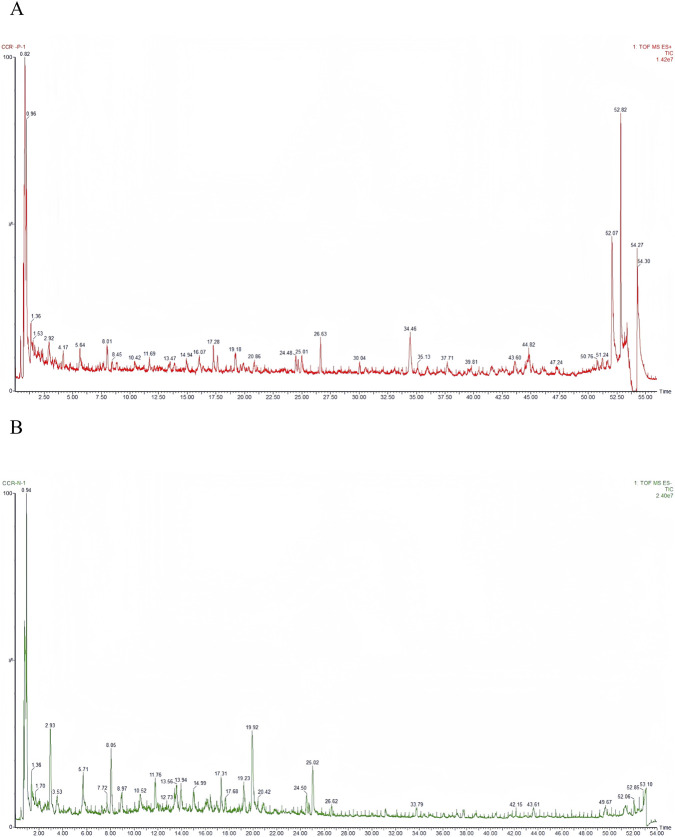
Total ion chromatogram of CCR extract components; **(A)** Positive ion mode; **(B)** Negative ion mode.

The CCR aqueous extract was subjected to full scan analysis in both positive and negative ion modes under the chromatographic and mass spectrometric conditions described above. The base peak ion chromatograms (BPI)exhibits good peak shape, peak intensity, and resolution, indicating that the chromatographic and MS conditions are suitable for sample analysis (Figure 1). A total of 153 components were identified, including 33 coumarins (e.g., phellopterin), 31 Terpenoids (e.g., Elemene), 26 flavonoids (e.g., luteolin), 2 organic acids (e.g., salicylic acid), 3 phenolics (e.g., Strictinin), 3 glycosides (e.g., narciclasine), 7 phenylpropanoids (e.g., ethyl caffeate), 2 tannins (e.g., rose tannin B), 1 anthraquinone (4-hydroxyrhein),1 phenolic acid (Gallic acid) and 44 others (Supplementary Table 1).

### 3.2 CCR used alone has a significant inhibitory effect on the proliferation of MCF-7 and T47D breast cancer cells

**FIGURE 2:**
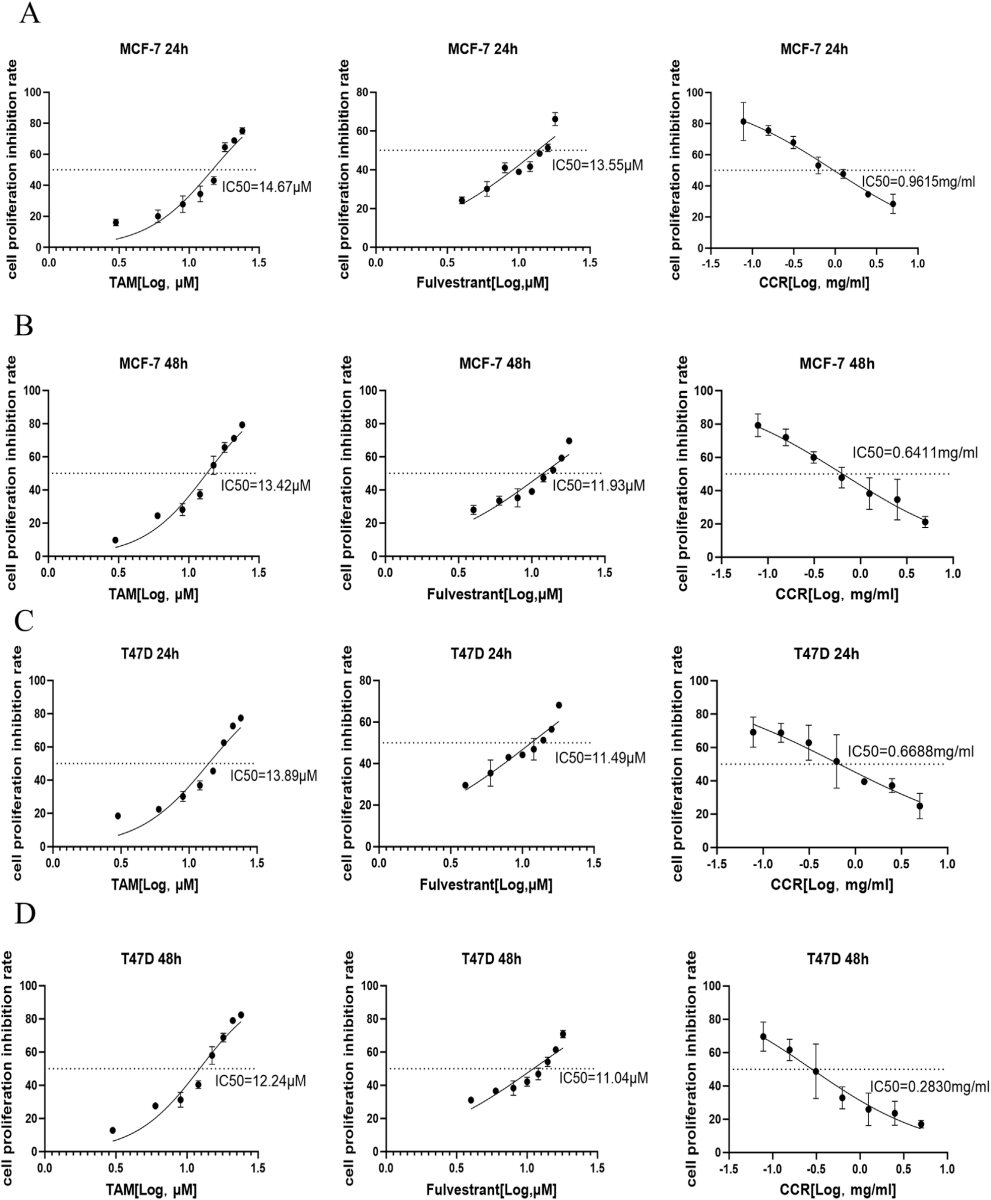
Inhibitory effects of CCR monotherapy on breast cancer MCF-7 and T47D cells. **(A)** Results after 24 h of treatment on MCF-7 cells. **(B)** Results after 48 h of treatment on MCF-7 cells. **(C)** Results after 24 h of treatment on T47D cells. **(D)** Results after 48 h of treatment on T47D cells, n = 3.

TAM, Ful, and CCR all inhibited the proliferation of MCF-7 and T47D breast cancer cells in a dose-dependent manner after 24 and 48 h of treatment. In MCF-7 cells, after 24 h of treatment, the IC50 values of TAM, Ful, and CCR were 14.67 μM, 13.55 μM, and 0.9615 mg/mL, respectively (Figure 2A); after 48 h, they were 13.42 μM, 11.93 μM, and 0.6411 mg/mL, respectively (Figure 2B). In T47D cells, after 24 h of treatment, the IC50 values of TAM, Ful, and CCR were 13.89 μM, 11.49 μM, and 0.6688 mg/mL, respectively (Figure 2C); after 48 h, the IC50 values were 12.24 μM, 11.04 μM, and 0.2830 mg/mL, respectively (Figure 2D).

### 3.3 The combination regimen exhibits no cytotoxicity in MCF-10A cells

**FIGURE 3:**
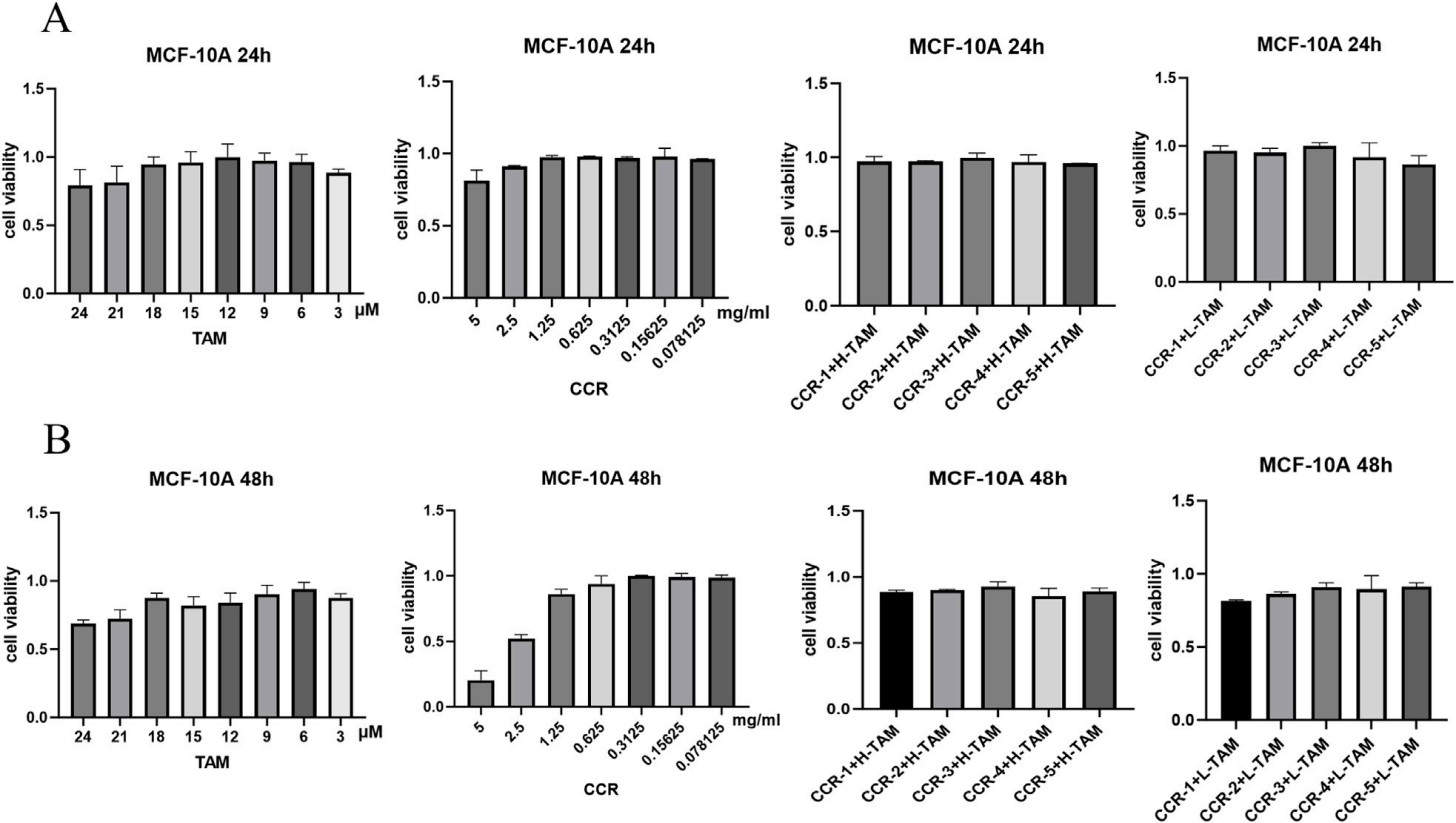
**(A)** Effects on MCF-10A cells after 24 h; **(B)** Effects on MCF-10A cells after 48 h; Data are presented as mean ± standard deviation (n = 3).

To evaluate the safety of the combined treatment, cytotoxicity was assessed in human breast epithelial MCF-10A cells after 24h and 48h. The combined doses of CCR and TAM have no significant effect on the proliferation of non-tumorigenic breast epithelial cells MCF-10A (Figures 3A,B).

### 3.4 CCR combined with TAM exerts synergistic anti-proliferative effects in MCF-7 and T47D cells

**FIGURE 4:**
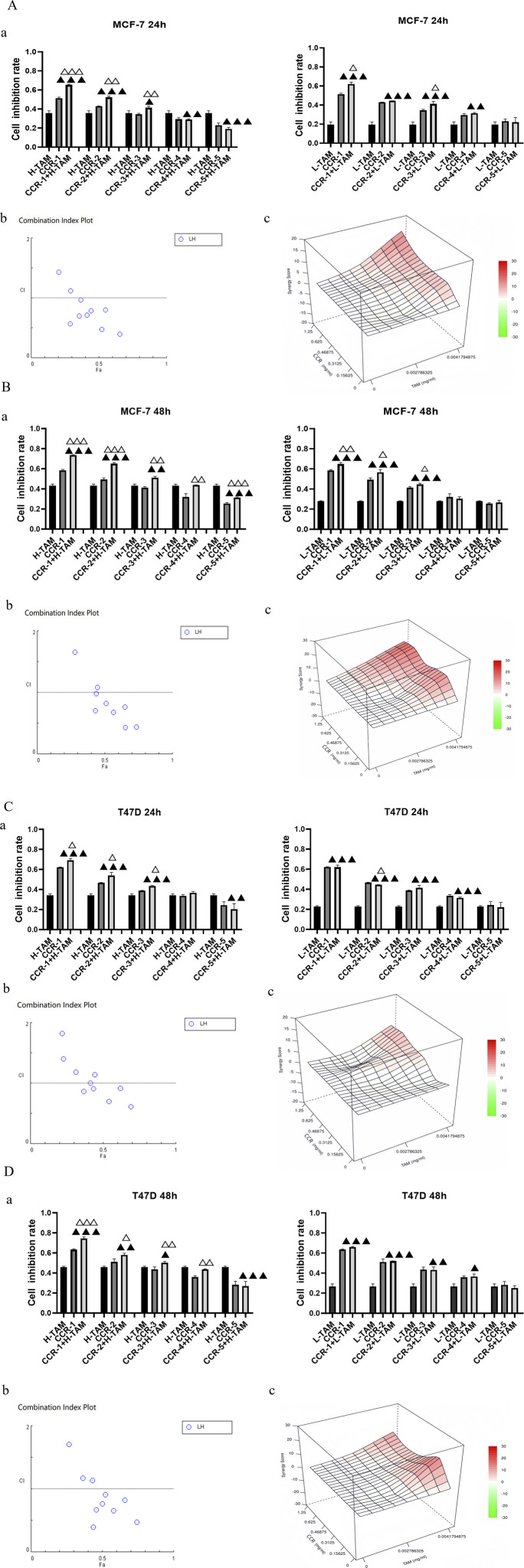
Results of the combined drug treatment on the antiproliferative effects in MCF-7 and T47D cells. **(A)** Cell inhibitory effects in MCF-7 cells after 24 h of treatment, **(a)** Combined treatment of H-TAM or L-TAM with different CCR formulations, **(b)** CI values after 24 h treatment, **(c)** Synergy scores after 24 h treatment. **(B)** Cell inhibitory effects in MCF-7 cells after 48 h of treatment, **(a)** Combined treatment of H-TAM or L-TAM with different CCR formulations, **(b)** CI values after 48 h treatment, **(C)** Synergy scores after 48 h treatment. **(C)** Cell inhibitory effects in T47D cells after 24 h of treatment, **(a)** Combined treatment of H-TAM or L-TAM with different CCR formulations, **(b)** CI values after 24 h treatment, **(c)** Synergy scores after 24 h treatment. **(D)** Cell inhibitory effects in T47D cells after 48 h of treatment, **(a)** Combined treatment of H-TAM or L-TAM with different CCR formulations, **(b)** CI values after 48 h treatment, **(c)** Synergy scores after 48 h treatment. ^△^ *p* < 0.05, ^△△^ *p* < 0.01, ^△△△^ *p* < 0.001, combined drug group compared with CCR group. ^▲^ *p* < 0.05, ^▲▲^ *p* < 0.01, ^▲▲▲^ *p* < 0.001,combined drug group compared with TAM group. Data are presented as mean ± standard deviation (n = 3).

In MCF-7 cells, two-way ANOVA revealed a significant interaction between CCR and TAM at 24 h (FDrug_A∗Drug_B = 24.884, *P* < 0.001). Combination treatments with high-dose TAM (H-TAM)plus CCR-1/2/3 and low-dose TAM(L-TAM)plus CCR-1/2/3/4 yielded significantly higher inhibition rates than monotherapy (Figure 4Aa). CompuSyn analysis confirmed significant synergism, with Confidence Interval (CI) values <1 at Fa >0.3 and a synergy score of 8.56 (Figures 4Ab,c). Following 48 h of treatment, the interaction was further strengthened (F = 68.328, *P* < 0.001), and most combinations produced markedly elevated inhibitory rates (Figures 4Ba). CI values remained <1 at Fa >0.4, and the synergy score increased to 11.58 (Figures 4Bb,c).

In T47D cells, a significant CCR–TAM interaction was detected at 24 h (F = 31.735, *P* < 0.001). Combined administration of H-TAM with CCR-1/2/3 and L-TAM with CCR-1/3 significantly increased inhibition rates (Figures 4Ca), accompanied by CI values <1 at Fa >0.4 and a synergy score of 5.32 (Figures 4Cb,c). After 48 h, the interaction remained significant (F = 49.092, *P* < 0.001), and most combinations showed markedly enhanced inhibitory effects (Figures 3Da). CI values were <1 at Fa >0.4, and the synergy score rose to 8.3 (Figures 4Db,c).

Median-effect analysis verified that CCR plus TAM mediated overall synergistic anti-proliferative effects, with CI values <1 over the Fa range of 0.3–0.8 in MCF-7 cells and 0.4–0.8 in T47D cells. By contrast, weak antagonism or sub-additivity was observed at low effect levels (Fa <0.3). On the basis of synergistic profiles and IC50 values, the combination of 0.46875 mg/mL CCR and 11.25 μM TAM (CCR-3+H-TAM) was chosen for subsequent mechanistic experiments.

### 3.5 The combined medication works by targeting and inhibiting the activities of ERα and SNCG, synergistically blocking estrogen-dependent proliferation signals

**FIGURE 5:**
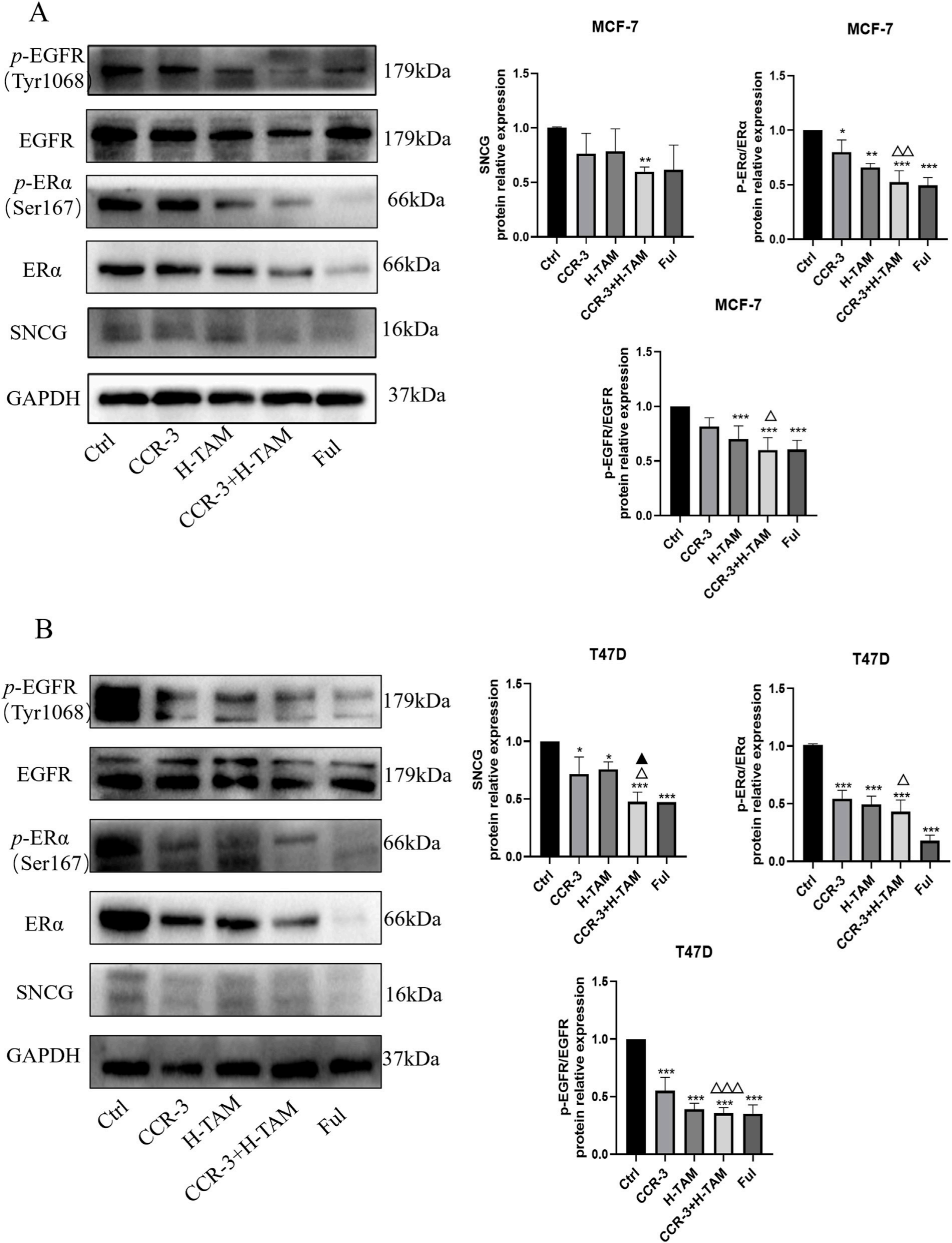
Effects of combined drug treatment on the expression levels of key molecules ERα and SNCG proteins. **(A)** Protein bands and relative quantification of proteins in MCF-7 cells. **(B)** Protein bands and relative quantification of proteins in T47D cells. ^#^ *p* < 0.05, ^##^ *p* < 0.01, ^###^ *p* < 0.001, compared with LV-EV group. *p < 0.05, **p < 0.01, ***p < 0.001,compared with LV-SNCG-OE group. ^△^ *p* < 0.05, ^△△^ *p* < 0.01, ^△△△^ *p* < 0.001, combined drug group compared with CCR group. ^▲^ *p* < 0.05, ^▲▲^ *p* < 0.01, ^▲▲▲^ *p* < 0.001,combined drug group compared with TAM group. Data are presented as mean ± standard deviation (n = 3).

Combination treatment significantly downregulated SNCG, *p*-ERα and *p*-EGFR protein levels in MCF-7 and T47D cells. Monotherapy with CCR, TAM or Ful also reduced the expression of these proteins, but the combination group exhibited a markedly stronger inhibitory effect on *p*-ERα and *p*-EGFR than CCR alone in MCF-7 cells (*P* < 0.05, *P* < 0.01) (Figure 5A). In T47D cells, combination treatment also more significantly downregulated SNCG, *p*-ERα and *p*-EGFR compared with CCR monotherapy (*P* < 0.05, *P* < 0.05, *P* < 0.01). Additionally, SNCG expression was significantly lower in the combination group than in the TAM group (*P* < 0.05) (Figure 5B). Collectively, combined therapy significantly repressed SNCG expression and synergistically inhibited ERα phosphorylation in MCF-7 and T47D cells, suggesting that inactivation of ER signaling may indirectly attenuate the oncogenic activity of SNCG. By co-regulating ERα and SNCG, the combination synergistically blocks estrogen-dependent proliferative signaling and suppresses compensatory EGFR activation.

### 3.6 SNCG and ERα may synergistically regulate the ERα signaling pathway through molecular interactions

**FIGURE 6:**
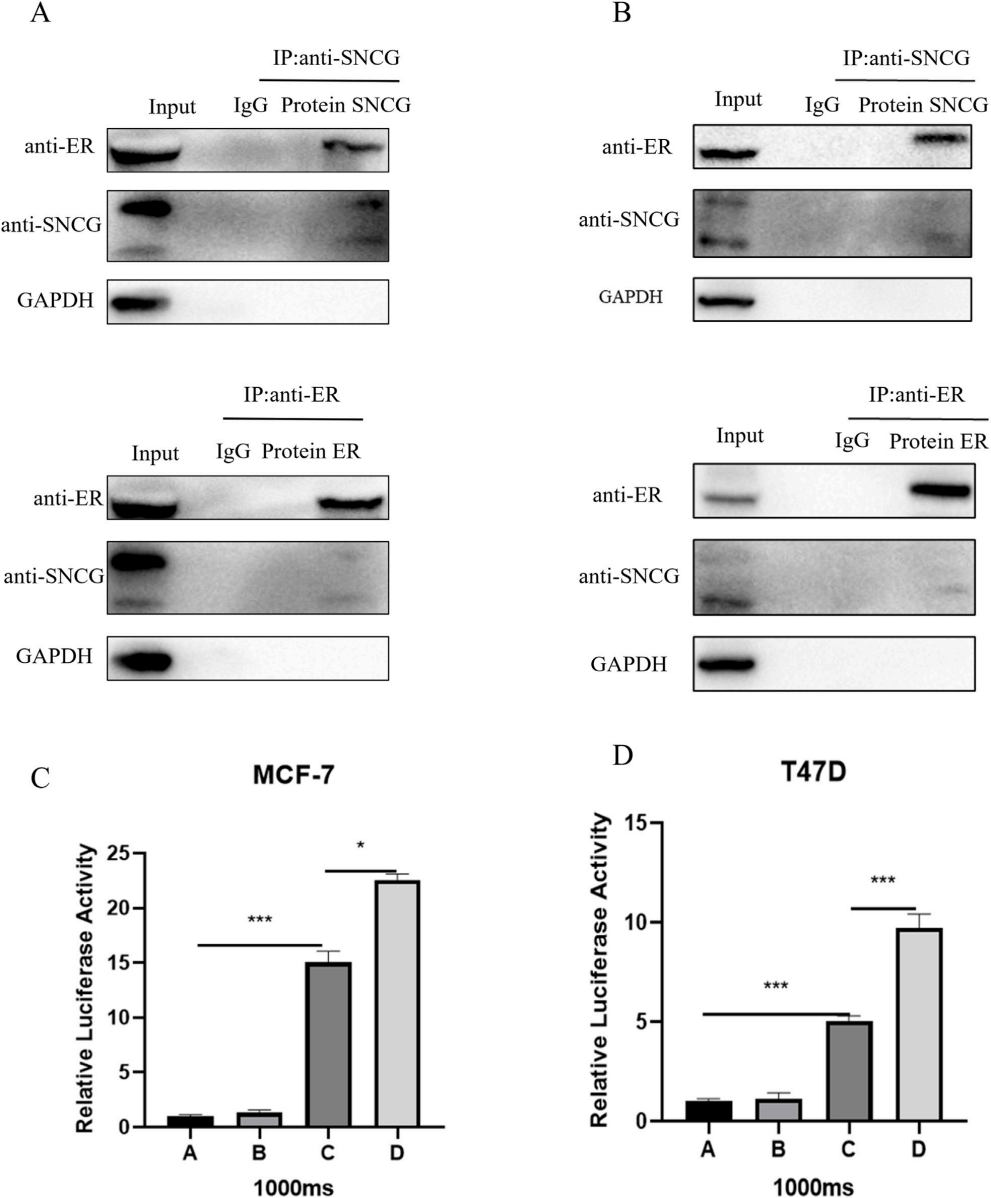
Interaction results between SNCG and ERα in breast cancer cells. **(A)** CoIP assay results in MCF-7 cells show interaction between SNCG and ERα. **(B)** CoIP assay results in T47D cells show interaction between SNCG and ERα. **(C)** The results of the dual luciferase reporter gene assay in MCF-7 cells showed that SNCG interacted with ERα. **(D)** The results of the dual luciferase reporter gene assay in T47D cells showed that SNCG interacted with ERα.

To further investigate the interaction between SNCG and ERα, CoIP and Dual-Lumi™ assays were performed. In the CoIP experiment (Figures 6A,B), when specific antibodies against either SNCG or ERα were used for immunoprecipitation, the other protein was effectively detected in the precipitated complex; no nonspecific binding was observed in the isotype IgG control group. The input samples showed sufficient expression of both SNCG and ERα, with consistent GAPDH expression as an internal control, indicating that the experimental system was stable and reliable. These results suggest that SNCG and ERα may form a protein complex in breast cancer cells. The Dual-Lumi™ assay (Figures 6C,D) confirmed that SNCG can specifically regulate the transcriptional activity of ERα as a transcription factor. The experimental results showed SNCG overexpression had no activation effect on a nonspecific promote rand the ERα promoter exhibited significant basal transcriptional activity; meanwhile in both cell lines tested, SNCG overexpression enhanced ERα promoter activity (*p* < 0.05 in MCF-7 cells, *p* < 0.001 in T47D cells).

### 3.7 Combination therapy exhibits potent anti-proliferative activity in SNCG-overexpressing MCF-7 and T47D cells

**FIGURE 7:**
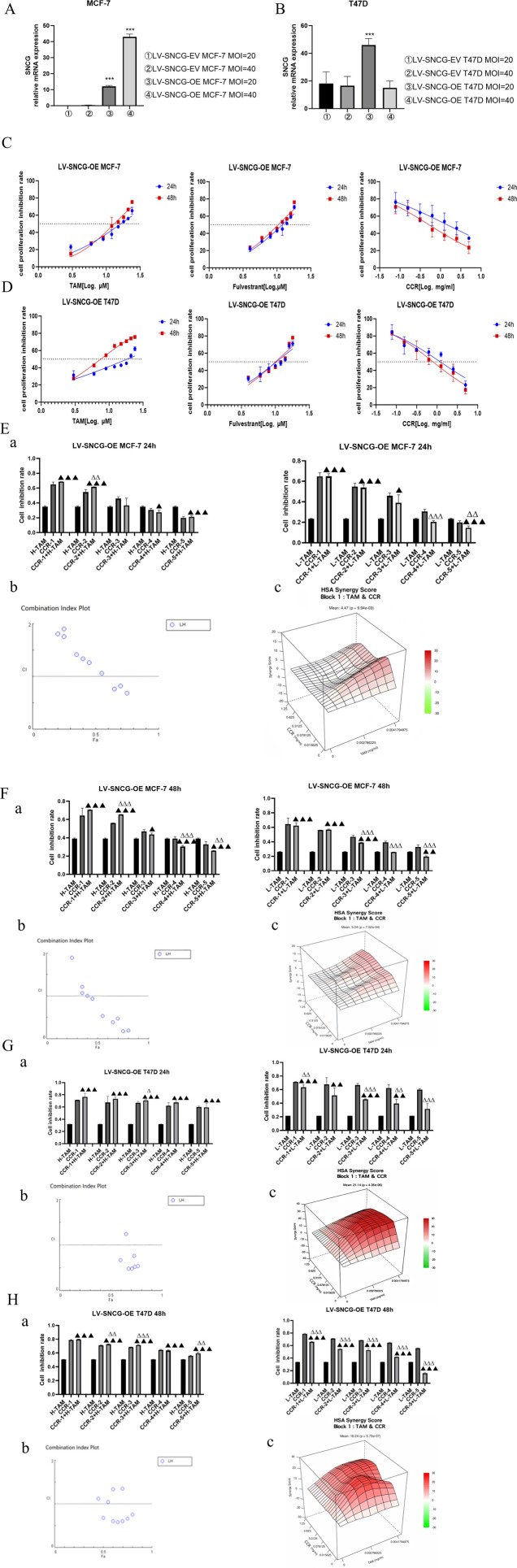
**(A)** mRNA expression level of SNCG in MCF-7 cells. **(B)** mRNA expression level of SNCG in T47D cells. **(C)** Effects of single-agent treatment on the proliferation of LV-SNCG-OE MCF-7 cells at 24 and 48 h. **(D)** Effects of single-agent treatment on the proliferation of LV-SNCG-OE T47D cells at 24 and 48 h. **(E)** Cell inhibitory effects in LV-MCF-7 cells after 24 h of treatment, **(a)** Combined treatment of H-TAM or L-TAM with different CCR formulations, **(b)** CI values after 24 h treatment, **(c)** Synergy scores after 24 h treatment. **(F)** Cell inhibitory effects in LV-MCF-7 cells after 48 h of treatment, **(a)** Combined treatment of H-TAM or L-TAM with different CCR formulations, **(b)** CI values after 48 h treatment, **(c)** Synergy scores after 48 h treatment. **(G)** Cell inhibitory effects in LV-T47D cells after 24 h of treatment, **(a)** Combined treatment of H-TAM or L-TAM with different CCR formulations, **(b)** CI values after 24 h treatment, **(c)** Synergy scores after 24 h treatment. **(H)** Cell inhibitory effects in LV-T47D cells after 48 h of treatment, **(a)** Combined treatment of H-TAM or L-TAM with different CCR formulations, **(b)** CI values after 48 h treatment, **(c)** Synergy scores after 48 h treatment. ^#^ *p* < 0.05, ^##^ *p* < 0.01, ^###^ *p* < 0.001, compared with LV-EV group. *p < 0.05, **p < 0.01, ***p < 0.001,compared with LV-SNCG-OE group. ^△^ *p* < 0.05, ^△△^ *p* < 0.01, ^△△△^ *p* < 0.001, combined drug group compared with CCR group. ^▲^ *p* < 0.05, ^▲▲^ *p* < 0.01, ^▲▲▲^ *p* < 0.001,combined drug group compared with TAM group. Data are presented as mean ± standard deviation (n = 3).

The efficiency of SNCG overexpression was detected by Real-time PCR, SNCG overexpression via lentiviral transduction increased 715-fold in MCF-7 cells (Figure 7A) and 2.5-fold in T47D cells (both *p* < 0.001) (Figure 7B).

CCR, TAM, and Ful all exhibited potent anti-proliferative effects on the proliferation (Figures 7C,D). Figure 7E shows that the IC50 values of TAM, Ful and CCR in LV-SNCG-OE MCF-7 cells were 16.4 μM, 12.02 μM and 1.457 mg/mL at 24 h, and 13.08 μM, 10.41 μM and 0.5185 mg/mL at 48 h, respectively. The IC50 values of TAM, Ful and CCR in LV-SNCG-OE T47D cells were 18.45 μM, 10.34 μM and 1.199 mg/mL at 24 h, and 7.537 μM, 10.06 μM and 0.7934 mg/mL at 48 h, respectively (Figure 7F). These data confirm that despite SNCG overexpression, all three drugs effectively inhibit cell proliferation, and prolonging the treatment time enhances their inhibitory effects.

In LV-SNCG-OE MCF-7 cells, Combination treatments with H-TAM plus CCR-1/2 yielded significantly higher inhibition rates than monotherapy (Figures 7Ea). CompuSyn analysis confirmed significant synergism, with CI values <1 at Fa >0.5 and a synergy score of 4.47 (Figures 7Eb,c). Following 48 h of treatment, the interaction was further strengthened (F = 46.293, *P* < 0.001), and most combinations produced markedly elevated inhibitory rates (Figures 7Fa). CI values remained <1 at Fa >0.5, and the synergy score increased to 5.04 (Figures 7Fb,c).

In LV-SNCG-OE T47D cells, a significant CCR–TAM interaction was detected at 24 h (F = 10.232, *P* < 0.001). Combined administration of H-TAM with CCR-1/2 significantly increased inhibition rates (Figures 7Ga), accompanied by CI values <1 at Fa <0.7 and a synergy score of 21.14 (Figures 7Gb,c). After 48 h, the interaction remained significant (F = 581.055, *P* < 0.001), and most combinations showed markedly enhanced inhibitory effects (Figures 7Ha). CI values were <1 at Fa <0.8, and the synergy score rose to 16.25 (Figures 7Hb,c), of note, the synergistic effects of CCR and TAM in LV-SNCG-OE T47D cells were predominantly concentrated at low-to-medium dose ranges, which was consistent with our previous findings. Integrating the CI, synergy scores, and two-way ANOVA results, a stable and overall synergistic effect was confirmed for the combination of CCR and TAM. Accordingly, the combination of CCR-2 (0.625 mg/mL) and high-dose TAM (11.25 μM) was selected for subsequent mechanistic studies.

### 3.8 The combined effect of CCR and TAM on the biological functions of SNCG-overexpressing cells

**FIGURE 8:**
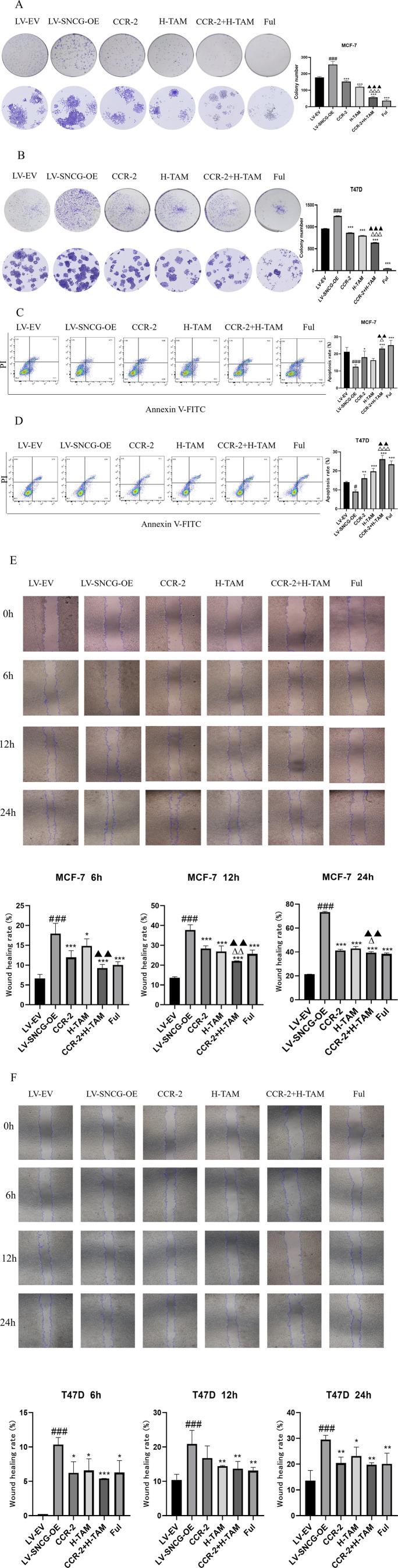
Effects of CCR plus TAM on colony formation **(A,B)**, apoptosis **(C,D)** and migration **(E,F)** in SNCG-overexpressing MCF-7 and T47D breast cancer cells. ^#^ *p* < 0.05, ^##^ *p* < 0.01, ^###^ *p* < 0.001, compared with LV-EV group. *p < 0.05, **p < 0.01, ***p < 0.001,compared with LV-SNCG-OE group. ^△^ *p* < 0.05, ^△△^ *p* < 0.01, ^△△△^ *p* < 0.001, combined drug group compared with CCR group. ^▲^ *p* < 0.05, ^▲▲^ *p* < 0.01, ^▲▲▲^ *p* < 0.001,combined drug group compared with TAM group. Data are presented as mean ± standard deviation (n = 3).

Further colony formation assays (Figures 8A,B) showed that SNCG overexpression significantly enhanced the cells’ colony-forming ability, with increased colony size and number in T47D cells. Compared to monotherapy, the combination of CCR and TAM significantly reduced both the number and size of colonies (both *p* < 0.001), with inhibitory effects markedly stronger than either drug alone. Annexin V-FITC/PI assay (Figures 8C,D)indicated that in SNCG-overexpressing MCF-7 and T47D cells, the total apoptosis rate in the combined CCR and TAM treatment group was significantly higher than that in each single-drug group (*p* < 0.05). Additionally, SNCG overexpression alone only inhibited apoptosis in MCF-7 cells (*p* < 0.001). Wound healing assay (Figures 8E,F)indicate that SNCG overexpression significantly enhances the migratory ability of MCF-7 and T47D cells, as evidenced by the cell healing rates at 6, 12, and 24 h in the LV-SNCG-OE group being significantly higher than those in the corresponding LV-EV group (*p* < 0.001). In both SNCG-overexpressing cell lines, treatment with CCR, TAM, combination therapy and Ful significantly inhibited cell migration compared to the LV-SNCG-OE group (*p* < 0.05). In MCF-7 cells, the migration inhibition effect of the combination therapy group was significantly stronger than that of the TAM or CCR monotherapy groups (*p* < 0.05). Although the differences between the combination therapy group and each monotherapy group in T47D cells did not reach statistical significance, the combination group consistently showed the strongest inhibitory trend at all time points. In summary, the combination of CCR and TAM clearly inhibits the migration of breast cancer cells overexpressing SNCG.

### 3.9 Combined use of CCR and TAM can inhibit SNCG, ERα signaling, and their downstream mTOR/AKT and *p*-ERK signaling pathway activities

**FIGURE 9:**
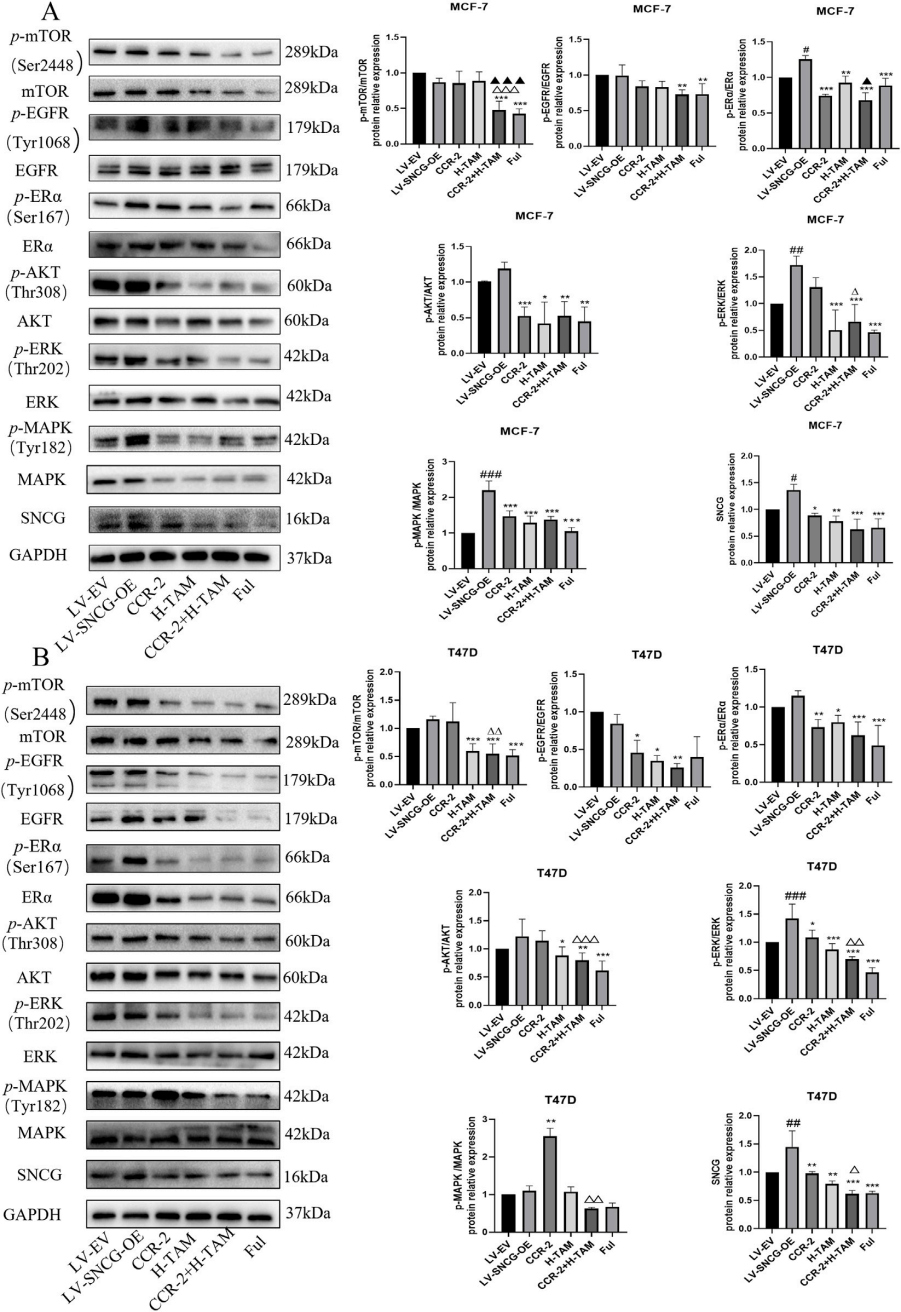
Effects of Combined Drug Treatment on the Expression Levels of Key Proteins ERα, SNCG, and Related Proteins in the PI3K/AKT/mTOR and EGFR/MAPK/ERK Signaling Pathways in SNCG-Overexpressing Breast Cancer Cells. **(A)** Protein bands and relative quantification of proteins in SNCG-overexpressing MCF-7 cells. **(B)** Protein bands and relative quantification of proteins in SNCG-overexpressing T47D cells. ^#^ *p* < 0.05, ^##^ *p* < 0.01, ^###^ *p* < 0.001, compared with LV-EV group. *p < 0.05, **p < 0.01, ***p < 0.001,compared with LV-SNCG-OE group. ^△^ *p* < 0.05, ^△△^ *p* < 0.01, ^△△△^ *p* < 0.001, combined drug group compared with CCR group. ^▲^ *p* < 0.05, ^▲▲^ *p* < 0.01, ^▲▲▲^ *p* < 0.001,combined drug group compared with TAM group. Data are presented as mean ± standard deviation (n = 3).

Western blot analysis revealed that CCR combined with TAM synergistically modulated multiple key signaling pathways (Figure 9). Under conditions without intervention, the levels of total AKT and *p*-AKT in SNCG-overexpressing cells showed no significant changes, while the level of *p*-ERK was significantly increased. This is consistent with previous reports (Zhuang et al., 2015), indicating that SNCG selectively activates the ERK pathway rather than the AKT pathway in ER + breast cancer cells. Notably, after combined treatment, the phosphorylation levels of AKT, ERK, and mTOR were significantly reduced (all *P* < 0.001), reflecting the broad-spectrum inhibitory effect of the combined treatment on multiple signaling pathways (Kinkade et al., 2008; Han et al., 2025). Additionally, the combination regimen also dramatically reduced the protein levels of SNCG and *p*-ERα. In T47D cells, whereas it reduced the levels of *p*-EGFR, *p*-mTOR, *p*-MAPK in MCF-7 cells (all *P* < 0.01). Interestingly, our data demonstrated that the regulation of the MAPK pathway by CCR is cell background-dependent, CCR synergized with SNCG to either increase or suppress *p*-MAPK expression in endogenously SNCG-positive T47D cells and endogenously SNCG-negative MCF-7 cells (Zhuang et al., 2015; Gupta et al., 2003), respectively. Collectively, these findings delineate the molecular mechanism by which the combined regimen exerts synergistic anti-tumor effects through concurrent regulating SNCG/*p*-ERα,mTOR/AKT and *p*-ERK pathway.

## 4 Discussion

Based on the established advantages of integrating traditional Chinese and Western medicine to optimise breast cancer treatment (Wu et al., 2025b; Man et al., 2024), as well as the fundamental anti-tumour properties of CCR confirmed in our previous research (Zhao Y. et al., 2024), this study further innovatively explores the synergistic therapeutic effects of CCR combined with TAM in ER + breast cancer. Unlike previous studies that only verified the basic efficacy of a single treatment drug, this research focuses on elucidating whether CCR can enhance the sensitivity of breast cancer cells to TAM treatment and improve its anti-tumour activity. Consistent with our core research hypothesis, current cell experiments indicate that the combination of CCR and TAM exhibits superior anti-proliferative effects compared to the use of CCR or TAM alone. Furthermore, we observed that MCF-7 and T47D cells display different sensitivities to CCR intervention, further supporting the unique bioactivity of CCR.

In this work, we compared the anti-proliferative capacities of CCR monotherapy, TAM monotherapy and their combined treatment in ER + breast cancer MCF-7 and T47D cells. Consistent with the general anti-tumor properties of the two agents, both CCR and TAM alone markedly suppressed the proliferation of the 2 cell lines. More importantly, combined administration of CCR and TAM exerted prominent synergistic anti-proliferative effects, which was far superior to the efficacy of either single agent. Our data also revealed that CCR displayed slightly stronger growth inhibition against T47D cells than MCF-7 cells at the concentration of 5 mg/mL after 48 h treatment. This cell line-dependent discrepancy was not fully consistent with a previous report (Vargas-Castro et al., 2023), which we speculate may be attributed to the higher sensitivity of T47D cells to the active ingredients contained in CCR.

To study whether there is a synergistic effect between the two drugs, we used two-way ANOVA, Compusyn software and SynergyFinder software to analyze the combined effect of CCR and TAM on the inhibition of proliferation in MCF-7 and T47D cells. CompuSyn software calculates confidence intervals using the Chou-Talalay equation, with data based on the median-effect equation, which is derived from the law of mass action principle (Ashton, 2015; Chou, 2010). This method has been well validated and widely reported in previous studies. It has been adopted to evaluate synergistic antitumor effects in multiple combination regimens, including PAK4 silencing combined with immunogenic phototherapy, acai berry extract combined with TAM or methotrexate, two Codonopsis radix extracts for pulmonary fibrosis, and GANT61 (a GLI1 inhibitor) combined with a PARP inhibitor against triple-negative breast cancer (Lu et al., 2023; Thornton et al., 2025; Pei et al., 2025; Mani et al., 2022). The data used for Compusyn software was entered into SynergyFinder software, which provides synergy maps based on mathematical models such as HSA and ZIP to analyze the interactions between two drugs (Zheng et al., 2022), visualize the data and calculate the maximum response of a single drug at the corresponding concentration under the expected combined effect (Foucquier and Guedj, 2015). The two mutually validate each other to jointly determine whether the interaction between CCR and TAM is antagonistic, additive, or synergistic. The results showed that in both MCF-7 and T47D cells, there was a significant interaction between the two drugs at both 24 and 48 h. Notably, the occasional occurrence of CI values greater than 1 at low Fa levels in combination index analysis is a common phenomenon in drug synergy studies, consistent with the effect level-dependent characteristics of the Chou-Talalay combination index theory (Chou, 2010; Chou and Talalay, 1984). Importantly, this observation does not contradict the overall synergistic trend of the combined regimen. Robust synergistic effects of CCR and TAM were consistently observed at clinically relevant effect levels (Fa = 0.5–0.8), and these findings were further validated by cell viability assays and SynergyFinder software. Collectively, these results highlight the importance of optimizing therapeutic doses to maximize the efficacy of combination therapy. Meanwhile, when applied to human breast epithelial MCF-10A cells, the combination treatment maintained stable cell viability. These experimental results indicate that the combination of TAM and CCR is safe. The combined treatment allowed for a reduction in the dosage of each drug, especially when synergy was significant. In our experiments, this combination reduced the doses of CCR and TAM to approximately 9.4% and 50%, respectively, of their single-agent doses. These findings have important clinical implications, as reducing drug dosages can help mitigate common toxicities and drug resistance during treatment. SNCG is abnormally expressed in breast cancer, especially in advanced stages (Lu et al., 2006), its expression is closely related to patient survival rates (Wu et al., 2007).

Research has indicated that SNCG, as Breast Cancer-Specific Gene 1 (BCSG1), can stimulate the transcriptional activity of ERα in breast cancer cells (Jiang et al., 2003; Liu et al., 2007). Further research has shown that knocking down endogenous SNCG can significantly inhibit ERα signaling; meanwhile, SNCG can also activate the ERK1/2 and mTOR signaling pathways by stabilizing ERα36. This process is closely related to TAM resistance in breast cancer (Shi et al., 2010). To further investigate the mechanism by which drug combination inhibits the proliferation of breast cancer cells, we evaluated the expression of ERα and SNCG-related proteins in ER + breast cancer under single and combined treatments. Our results showed that treatment with CCR alone or in combination effectively suppressed the expression of SNCG, *p*-ERα, and *p*-EGFR proteins in both tested cell lines. These findings are consistent with previously reported studies, indicating that inhibiting SNCG expression can reduce the proliferation of breast cancer cells (He et al., 2014).

To thoroughly assess the precise role of SNCG in the two detected breast cancer cell lines and whether it interacts with ERα, we constructed an SNCG overexpression cell model. This study demonstrated the interaction between SNCG and ERα through immunoprecipitation. In both cell models examined, SNCG specifically bound to ERα. These results are consistent with previous studies (Lu et al., 2013). In the ERα antibody-mediated precipitation group, although the detected signal intensity of SNCG was relatively weak, its binding specificity was not affected. This phenomenon may be due to a stronger interaction between ERα and the heat shock proteins Hsp70 or Hsp90, which have been shown to bind to estrogen receptors and regulate ER-mediated cell proliferation (Dhamad et al., 2016), it may also be related to potential factors such as differences in the intracellular abundance of the target protein, antibody binding efficiency, or complex stability. Dual-luciferase reporter gene assays further revealed that SNCG overexpression significantly enhances ERα promoter activity, suggesting that SNCG overexpression enhanced ERα promoter activity. Western blot experiments showed that SNCG overexpression did not induce changes in either ERα phosphorylation or total protein levels, which is consistent with previous reports (Jiang et al., 2004; Jiang et al., 2003). However, combined drug treatment synergistically downregulates SNCG and ERα expression, the underlying mechanism may involve TAM-mediated suppression of ERα expression (Huang et al., 2015) and induction of SNCG degradation by one component in CCR.

Literature reports that the function of SNCG in breast cancer is closely related to the ERK and AKT/mTOR pathways (Liang et al., 2015; Zhuang et al., 2015). MAPK/ERK is a key signaling axis that regulates cell proliferation, survival and drug resistance (Kizilboga et al., 2019),its activation is related to the promotion of breast cancer cell growth (Song et al., 2015), EGFR is a major receptor tyrosine kinase involved in MAPK/ERK signaling and the development of breast cancer (Arteaga, 2002). The interaction between ERα and the EGFR signaling pathway in breast cancer has been reported in the literature (Britton et al., 2006; Li et al., 2020b). This study shows that CCR significantly inhibits SNCG protein expression, while TAM exhibits a synergistic effect only when used in combination, suggesting that CCR primarily acts by directly targeting SNCG transcription or stability, whereas TAM relies more on feedback regulation following interference with ERα signaling. This indicates that combination therapy can reverse the activation of the above pathways through multiple targets, significantly downregulating *p*-EGFR, blocking the initiation signals of EGFR/MAPK, and reducing *p*-ERK, inhibiting MAPK/ERK further decreases ERα phosphorylation, forming a negative feedback loop that blocks the compensatory activation of ERα-independent proliferative signals. The PI3K/AKT/mTOR pathway serves as a core pathway in the development and drug resistance of ER + breast cancer (Alves and Ditzel, 2023). In SNCG-overexpressing breast cancer cells, combination treatment significantly downregulated *p*-mTOR and *p*-AKT. Notably, the drug effects were cell-specific. The inhibitory effect of the combination treatment on *p*-EGFR was significant in MCF-7 cells but not obvious in T47D cells. This may be related to the loss of PTEN in T47D cells, which allows continued activation of the PI3K pathway to maintain survival even after EGFR inhibition (Citi et al., 2018).

Combination therapy can significantly inhibit cell proliferation, with a more pronounced synergistic effect observed in SNCG-overexpressing cells. This may be related to the higher affinity of certain active components in CCR for the SNCG protein, allowing them to more readily accumulate in target regions within the SNCG-overexpressing model. This, in turn, enhances the dissociation efficiency of the ERα/SNCG complex, demonstrating the advantage of multi-target regulation by traditional Chinese medicine formulas. Among the cell lines tested, T47D cells showed a more prominent synergistic response to the combination therapy, which aligns with the trend observed in our previous experiments but differs from the findings reported by Pawlik et al. (2016),This may be related to PTEN loss leading to constitutive activation of the PI3K/AKT/mTOR pathway, making it more sensitive to multi-targeted interventions. Combination therapy can significantly inhibit the colony-forming ability of SNCG-overexpressing cells, increase the overall apoptosis rate, and reduce cell migration ability, confirming its antitumor activity from multiple dimensions. There are obvious cell line-specific differences in functional responses. In T47D cells, SNCG overexpression more significantly promotes colony formation, whereas in MCF-7 cells, SNCG overexpression more notably mediates apoptosis inhibition. This may be related to the high expression of EGFR or imbalance of Bcl-2 family proteins in MCF-7 cells (Witters et al., 2007). Meanwhile, the combined drug treatment exhibited a superior synergistic inhibitory effect on cell migration in MCF-7 cells, which may be related to the baseline differences in EMT-related markers between the 2 cell lines. This suggests that cell type specificity may influence variations in drug response, potentially due to inherent heterogeneity in cellular signaling pathways. Future studies on combination therapies should optimize research protocols by selecting different cell models according to specific needs.

## 5 Conclusion

This study further clarifies the anti-cancer effects of the liver-soothing and depression-relieving therapy, it enhances the activity of TAM against MCF-7 and T47D cell lines by regulating SNCG to inhibit cell migration and formation, inducing apoptosis, and regulating the activity AKT/mTOR and *p*-ERK pathways, as well as ERα signaling. According to our research findings, CCR can be used in combination with TAM to treat breast cancer, enhancing efficacy while allowing for a reduced TAM dosage, thereby minimizing its toxicity and side effects.

## Data Availability

The raw mass spectrometry data generated in this study are available in Figshare at https://doi.org/10.6084/m9.figshare.32790417.
