# Supplementary material for: Shu Gan Jie Yu herbal combination enhances tamoxifen sensitivity in breast cancer cells via modulation of the SNCG/p-ERα,mTOR /AKT and p-ERK pathway
Source: Front Pharmacol. 2026 Jul 27;17:1868856. doi: 10.3389/fphar.2026.1868856 (PMC13454320; doi:10.3389/fphar.2026.1868856)
Supplement: Supplementary file 1 [file DataSheet1.docx]

***Supplementary Material***

Supplementary Table 1 Analysis of CCR components based on UPLC-Q-TOF-MS technology

| Serial number | English name | Theoretical value  m/z | Measured value  m/z | Error /ppm | RT  /min | Addition  Method | Molecular formula | MS/MS | Category |
| --- | --- | --- | --- | --- | --- | --- | --- | --- | --- |
| 1 | L-Glutamic acid | 147.0531 | 148.0604 | -0.1 | 0.87 | [M+H]^+^ | C_5_H_9_NO_4_ | 148.06225、130.04904、113.02164 | Other categories |
| 2 | sucrose | 342.1155 | 365.1047 | -1.9 | 0.91 | [M+Na]^+^ | C_12_H_22_O_11_ | 343.12241、325.11179、307.10185、145.04960 | Glycosides |
| 3 | Nobiletin | 402.1275 | 403.1348 | -9.8 | 0.95 | [M+H]^+^ | C_21_H_22_O_8_ | 403.13199、373.12627 、137.06955、107.06171 | Flavonoids |
| 4 | Imperatorin | 270.0882 | 293.0774 | -3.5 | 1.43 | [M+Na]^+^ | C_16_H_14_O_4_ | 223.07210、215.03418、203.03408、201.01581、 187.03892、185.02343、175.03726、157.02966、 147.04586、145.02830 | Coumarins |
| 5 | Isoimperatorin | 270.0882 | 293.0774 | -3.5 | 1.43 | [M+Na]^+^ | C_16_H_14_O_4_ | 215.03478、203.03408、201.01581、187.03892、 185.02343、175.03776、173.02286、157.02966、 145.02830 | Coumarins |
| 6 | Gallic acid | 170.021 | 171.0283 | -3 | 2.91 | [M+H]^+^ | C_7_H_6_O_5_ | 171.02941、153.01777、127.03871、125.02282 、123.00207、109.02810 | phenolic acid |
| 7 | Kojic acid | 142.0267 | 143.0339 | 0.4 | 5.64 | [M+H]^+^ | C_6_H_6_O_4_ | 143.03323、125.02281 | Other categories |
| 8 | vanillic acid | 168.0432 | 191.0324 | 5 | 5.65 | [M+Na]^+^ | C_8_H_8_O_4_ | 153.01814、151.03860、143.03323、127.03790 、125.02281、109.02807 | Organic acids |
| 9 | Fraxetin | 208.0365 | 209.0438 | -3 | 5.65 | [M+H]^+^ | C_10_H_8_O_5_ | 209.04325、191.03357、165.01714、163.03849 、109.02807、105.03341 | Coumarins |
| 10 | apigenin | 270.053 | 271.0603 | 0.6 | 5.76 | [M+H]^+^ | C_15_H_10_O_5_ | 255.02853、245.04360 、227.03477、119.04846 | Flavonoids |
| 11 | Eriodictyol | 288.0627 | 289.07 | -2.4 | 5.76 | [M+H]^+^ | C_15_H_12_O_6_ | 289.07011、273.03915 、245.04360、139.03886、135.04424、119.04846 | Flavonoids |
| 12 | ammiol | 276.0636 | 277.0709 | 0.8 | 8.59 | [M+H]^+^ | C_14_H_12_O_6_ | 277.07353、259.06461、193.04756、137.02369 | Other categories |
| 13 | salicylic acid | 138.0313 | 139.0385 | -3.1 | 10.55 | [M+H]^+^ | C_7_H_6_O_3_ | 139.03864、111.04361 | Organic acids |
| 14 | L-Epicatechin | 290.079 | 291.0863 | 0 | 10.55 | [M+H]^+^ | C_15_H_14_O_6_ | 291.08502、289.07179、245.04413、165.05491、 147.04389、139.03864、137.02239、123.04346 、111.04361 | Other categories |
| 15 | chlorogenic acid | 354.0939 | 355.1012 | -3.4 | 10.85 | [M+H]^+^ | C_16_H_18_O_9_ | 355.10208 、133.02494 | Phenylpropanoids |
| 16 | Scopolin | 354.0939 | 355.1012 | -3.4 | 10.85 | [M+H]^+^ | C_16_H_18_O_9_ | 355.10208 、193.04927、 147.04425 | Coumarins |
| 17 | Isoscopoletin | 192.042 | 193.0492 | -1.5 | 11.72 | [M+H]^+^ | C_10_H_8_O_4_ | 193.04913 、175.03840 、163.03910、 147.04349 、133.02866 、105.03380 | Coumarins |
| 18 | Scopoletin | 192.042 | 193.0492 | -1.5 | 11.72 | [M+H]^+^ | C_10_H_8_O_4_ | 175.03840 、163.03910 、133.02866 | Coumarins |
| 19 | 5,7-DIHYDROXY-4-METHYLCOUMARIN | 192.042 | 193.0492 | -1.5 | 11.72 | [M+H]^+^ | C_10_H_8_O_4_ | 193.04913 、191.03391 、175.03840、 165.05255 | Coumarins |
| 20 | 8-Hydroxybergapten | 232.0355 | 233.0428 | -7.3 | 11.74 | [M+H]^+^ | C_12_H_8_O_5_ | 233.04392 、205.04845 、191.03391、187.03789 177.05448 、163.03910 | Coumarins |
| 21 | Strictinin | 634.0859 | 635.0931 | 8.3 | 11.98 | [M+H]^+^ | C_27_H_22_O_18_ | 617.07824 、467.08305 、447.05752、 277.03385 、155.03292、153.01811、127.03815 、125.02300 109.02772 | Phenols |
| 22 | Isocoumarin | 146.0367 | 147.0439 | -0.9 | 12.21 | [M+H]^+^ | C_9_H_6_O_2_ | 147.04388、 119.04863 、117.03321 | Coumarins |
| 23 | casuarictin | 936.0819 | 937.0891 | -5.4 | 13.39 | [M+H]^+^ | C_41_H_28_O_26_ | 767.07337、153.01799、125.02296 | Tannins |
| 24 | tellimagrandin I | 786.0882 | 787.0955 | -4.3 | 13.54 | [M+H]^+^ | C_34_H_26_O_22_ | 787.09615、769.08013 、617.07580 、599.06387 447.05752 、155.03292 、153.01811 、127.03906、125.02281 | Other categories |
| 25 | rugosin B | 954.0919 | 955.0991 | -5.9 | 13.89 | [M+H]^+^ | C_41_H_30_O_27_ | 153.01796、 127.03887、 125.02279 | Tannins |
| 26 | L-ASPARTIC ACID | 133.0375 | 134.0447 | -0.4 | 13.93 | [M+H]^+^ | C_4_H_7_NO_4_ | 134.04431、 116.03283 | Other categories |
| 27 | (-)-TERPINEN-4-OL | 154.1355 | 155.1428 | -1.8 | 16.44 | [M+H]^+^ | C_10_H_18_O | 155.14326 、137.13196 | Terpenoids |
| 28 | (1alpha,2alpha,5alpha)-2-methyl-5-(1-methylethyl)bicyclo[3.1.0]hexan-2-ol | 154.1355 | 155.1428 | -1.8 | 16.44 | [M+H]^+^ | C_10_H_18_O | 155.14326 、137.13196 | Other categories |
| 29 | (l)-alpha-terpineol | 154.1355 | 155.1428 | -1.8 | 16.44 | [M+H]^+^ | C_10_H_18_O | 155.14326 、137.13196 | Terpenoids |
| 30 | 3,7-dimethyl-1,7-Octadien-3-ol | 154.1355 | 155.1428 | -1.8 | 16.44 | [M+H]^+^ | C_10_H_18_O | 155.14326 、137.13196 | Other categories |
| 31 | Nerol | 154.1355 | 155.1428 | -1.8 | 16.44 | [M+H]^+^ | C_10_H_18_O | 155.14326 、137.13196 | Other categories |
| 32 | Isofraxidin | 222.0551 | 223.0623 | 10 | 17.62 | [M+H]^+^ | C_11_H_10_O_5_ | 223.06223 、177.01841 、175.03882、 151.03843 137.02345 、121.02776 、107.04900 | Coumarins |
| 33 | Fraxinol | 222.0551 | 223.0623 | 10 | 17.62 | [M+H]^+^ | C_11_H_10_O_5_ | 223.06223 、151.03843 、137.02345、121.02776 | Coumarins |
| 34 | Isorhamnetin 3-O-neohesperidoside | 624.1684 | 625.1757 | -1 | 17.66 | [M+H]^+^ | C_28_H_32_O_16_ | 625.17452 、317.06069 | Other categories |
| 35 | Kaempferol | 286.0476 | 287.0549 | -0.6 | 19.2 | [M+H]^+^ | C_15_H_10_O_6_ | 287.05454 、259.02143 、257.00749、241.01038 、231.02789 、219.02817、213.05466 、203.03297 、153.01784、137.02318 、135.04389 、133.02978 121.02825 、111.00680 | Flavonoids |
| 36 | Luteolin | 286.0476 | 287.0549 | -0.6 | 19.2 | [M+H]^+^ | C_15_H_10_O_6_ | 259.06285 、241.04934 、231.06510、 229.04864 、203.03297、201.01507 、191.03379、153.01784 、137.02318 | Flavonoids |
| 37 | RUTIN | 610.152 | 611.1593 | -2.2 | 19.2 | [M+H]^+^ | C_27_H_30_O_16_ | 449.10679 、287.05454 、153.01784、145.02793、 137.02318 、133.02978、109.02833 | Flavonoids |
| 38 | catenarin | 286.0476 | 287.0549 | -0.6 | 19.2 | [M+H]^+^ | C_15_H_10_O_6_ | 287.05454 、247.02360 、241.04934、219.02871 203.03297 、191.03379、 165.01745 、137.02318 | Anthraquinones |
| 39 | Demethoxycapillarisin | 286.0476 | 287.0549 | -0.6 | 19.2 | [M+H]^+^ | C_15_H_10_O_6_ | 287.05454 、257.00749 、241.04934、231.02789 、219.02871、 203.03297、153.01784 、137.02318 、135.04389、 133.02978、 109.02833 | Other categories |
| 40 | ETHYL CAFFEATE | 208.0732 | 209.0805 | -1.7 | 19.27 | [M+H]^+^ | C_11_H_12_O_4_ | 209.08022 、191.07022 、163.03757、135.04389 | Phenylpropanoids |
| 41 | Isomeranzin | 260.1023 | 261.1096 | -9.8 | 19.69 | [M+H]^+^ | C_15_H_16_O_4_ | 161.05889、 159.04246 、145.06496、131.04914 、121.02732 | Coumarins |
| 42 | Meranzin | 260.1023 | 261.1096 | -9.8 | 19.69 | [M+H]^+^ | C_15_H_16_O_4_ | 161.05889 、159.04246 、131.0414 、121.02732 | Coumarins |
| 43 | Neoeriocitrin | 596.1709 | 597.1782 | -5.4 | 19.83 | [M+H]^+^ | C_27_H_32_O_15_ | 289.07010 、259.06560、 163.03792、153.01774 | Flavonoids |
| 44 | Suberosin | 244.1096 | 245.1168 | -1.6 | 19.87 | [M+H]^+^ | C_15_H_16_O_3_ | 245.11634 、175.03878 、173.02235、163.03792 、145.02767 | Coumarins |
| 45 | Narcissin | 624.1676 | 625.1749 | -2.3 | 20.19 | [M+H]^+^ | C_28_H_32_O_16_ | 433.11021、 317.06502 、301.03299、 285.03870、 273.03572、 271.06185、 245.04263 | Glycosides |
| 46 | Isovitexin | 432.1045 | 433.1118 | -2.7 | 20.53 | [M+H]^+^ | C_21_H_20_O_10_ | 301.07061 、283.06159 | Flavonoids |
| 47 | Prunetin | 284.0677 | 285.075 | -2.7 | 20.8 | [M+H]^+^ | C_16_H_12_O_5_ | 243.06586、199.03399 、121.02770 、119.04942 | Flavonoids |
| 48 | 3,4,7-trimethoxycoumarin | 236.0677 | 237.075 | -3.2 | 20.8 | [M+H]^+^ | C_12_H_12_O_5_ | 237.07483 、191.03417、 177.05418、107.04884 | Coumarins |
| 49 | 5,6,7-Trimethoxycoumarin | 236.0677 | 237.075 | -3.2 | 20.8 | [M+H]^+^ | C_12_H_12_O_5_ | 237.07483 、177.05418 、163.03791、149.05990 、121.02770 、107.04884 | Coumarins |
| 50 | 7-Methoxycoumarin | 176.0468 | 177.0541 | -3 | 20.8 | [M+H]^+^ | C_10_H_8_O_3_ | 177.05418 、149.05990 、147.04402、131.04800、121、02770 、119.04942、107.04884 | Coumarins |
| 51 | Ayapanin | 176.0468 | 177.0541 | -3 | 20.8 | [M+H]^+^ | C_10_H_8_O_3_ | 177.05418、 149.05990、 147.04412、131.04800、 121.02770、 119.04942、 107.04884 | Coumarins |
| 52 | khellin | 260.0676 | 261.0749 | -3.2 | 20.8 | [M+H]^+^ | C_14_H_12_O_5_ | 243.06586 、217.04861、 177.05418 | Other categories |
| 53 | Quercetin | 302.0422 | 303.0495 | -1.5 | 20.86 | [M+H]^+^ | C_15_H_10_O_7_ | 303.04936 、285.03849 、275.05627、 257.04374 247.06036、 245.04309 、229.04878、 217.04861 165.01715 、153.01751 、137.02288、 121.02770 111.04348 | Flavonoids |
| 54 | Hirsutrin | 464.0942 | 465.1015 | -2.7 | 20.86 | [M+H]^+^ | C_21_H_20_O_12_ | 465.10515、 287.05439 、285.03849、 247.06036、 245.04309 、153.01751、 137.02288、 123.04420 、109.02832 | Flavonoids |
| 55 | Isoquercetin | 464.0942 | 465.1015 | -2.7 | 20.86 | [M+H]^+^ | C_21_H_20_O_12_ | 465.10515、 287.05439、 285.03849、247.06036 245.04309 、153.01751 、137.02288 、111.04348、 109.02832 | Flavonoids |
| 56 | Lonicerin | 594.1574 | 595.1647 | -1.8 | 20.88 | [M+H]^+^ | C_27_H_30_O_15_ | 595.16056、 449.10811、 287.05439 | Flavonoids |
| 57 | Quercetin 3-O-glucoside-7-O-rhamnoside | 594.1574 | 595.1647 | -1.8 | 20.88 | [M+H]^+^ | C_27_H_30_O_15_ | 465.10515 、449.10811、 303.04936、 287.05439 、285.03849 | Flavonoids |
| 58 | Cynaroside | 448.0995 | 449.1068 | -2.3 | 20.9 | [M+H]^+^ | C_21_H_20_O_11_ | 449.10811、 287.05439 、285.03849、179.03352 | Glycosides |
| 59 | QUERCITRIN | 448.0995 | 449.1068 | -2.3 | 20.9 | [M+H]^+^ | C_21_H_20_O_11_ | 449.10811 、287.05439 、285.03849、247.06036 、245.04309 、153.01751、137.02288 、123.04420 、111.04348、109.02833 | Flavonoids |
| 60 | Astragalin | 448.0995 | 449.1068 | -2.3 | 20.9 | [M+H]^+^ | C_21_H_20_O_11_ | 449.10811 、287.05439 、285.03849、 257.04374、 245.04309 、153.01751、 137.02288 、121.02770 、107.04884 | Flavonoids |
| 61 | Vicenin-2 | 594.1577 | 595.165 | -1.2 | 21.95 | [M+H]^+^ | C_27_H_30_O_15_ | 595.16517 、445.11236 | Flavonoids |
| 62 | Naringenin | 272.0679 | 273.0751 | -2.3 | 23.17 | [M+H]^+^ | C_15_H_12_O_5_ | 273.07453 、147.04437 | Flavonoids |
| 63 | γ-calacorene | 200.156 | 201.1633 | -2.4 | 23.17 | [M+H]^+^ | C_15_H_20_ | 201.16453 、173.13240 、159.11646、 145.10014 、143.08481 、131.08512、117.06960、 105.06962 | Terpenoids |
| 64 | scoparone | 206.0575 | 207.0648 | -2 | 23.53 | [M+H]^+^ | C_11_H_10_O_4_ | 207.06461 、206.05745 、192.03694、191.03332 、179.06817 、164.04125、163.03830 、162.03004 、151.07476、148.05068 、147.04275 、146.03574 145.02692 、136.05380 、135.04402、134.03627、 133.02408 、121.06466、119.04862 、118.04084 、117.03274、108.05506 、107.04884 | Coumarins |
| 65 | Isorhamnetin | 316.0594 | 317.0667 | 3.6 | 24.48 | [M+H]^+^ | C_16_H_12_O_7_ | 317.06336 、263.05533、 245.04354、231.02696 、219.02722、 217.04851、 153.01788 、135.04295、111.04389 | Flavonoids |
| 66 | Hesperetin | 302.0787 | 303.086 | -1 | 24.5 | [M+H]^+^ | C_16_H_14_O_6_ | 303.08615、 285.07633、 245.04354、179.03372 、177.05412 、175.03622、 163.03990 、153.01788 、151.03956、149.05950、 135.04295、 123.04310 | Flavonoids |
| 67 | 4-METHYLUMBELLIFERONE | 176.0467 | 177.054 | -3.6 | 24.5 | [M+H]^+^ | C_10_H_8_O_3_ | 177.05412 、151.03956、 135.04295、 111.04389 、109.02743 、107.04986 | Coumarins |
| 68 | chrysoeriol | 300.0632 | 301.0705 | -0.5 | 24.69 | [M+H]^+^ | C_16_H_12_O_6_ | 301.07040、 257.04358 、203.03272、153.01755 | Flavonoids |
| 69 | Diosmetin | 300.0632 | 301.0705 | -0.5 | 24.69 | [M+H]^+^ | C_16_H_12_O_6_ | 301.07040 、257.04358 、203.03272、 187.03976 153.01755 | Flavonoids |
| 70 | Coumarin | 146.0367 | 147.044 | -0.4 | 25.35 | [M+H]^+^ | C_9_H_6_O_2_ | 147.04365 、121.02686 | Coumarins |
| 71 | Sabinene hydrate | 154.1372 | 177.1265 | 8.4 | 25.91 | [M+Na]^+^ | C_10_H_18_O | 177.12580 、111.08078 | Other categories |
| 72 | beta-elemene | 204.1863 | 227.1756 | -6.4 | 26.63 | [M+Na]^+^ | C_15_H_24_ | 135.11656 、107.08562 | Terpenoids |
| 73 | β-elemene | 204.1863 | 227.1756 | -6.4 | 26.63 | [M+Na]^+^ | C_15_H_24_ | 135.11656 、107.08562 | Terpenoids |
| 74 | Elemene | 204.1863 | 227.1756 | -6.4 | 26.63 | [M+Na]^+^ | C_15_H_24_ | 135.11656 、107.08562 | Terpenoids |
| 75 | rotundene | 204.1863 | 227.1756 | -6.4 | 26.63 | [M+Na]^+^ | C_15_H_24_ | 227.17582 、147.11815 | Other categories |
| 76 | Bicyclo[5,2,0]nonane,2-methylene-4,8,8-trimethyl-4-vinyl | 204.1863 | 227.1756 | -6.4 | 26.63 | [M+Na]^+^ | C_15_H_24_ | 227.17582 、147.11815、 107.08562 | Other categories |
| 77 | copaene | 204.1863 | 227.1756 | -6.4 | 26.63 | [M+Na]^+^ | C_15_H_24_ | 227.17582 、135.11656 | Terpenoids |
| 78 | cyperene | 204.1863 | 227.1756 | -6.4 | 26.63 | [M+Na]^+^ | C_15_H_24_ | 227.17582 、147.11815 | Other categories |
| 79 | isodaucene | 204.1863 | 227.1756 | -6.4 | 26.63 | [M+Na]^+^ | C_15_H_24_ | 227.17582、135.11656 | Terpenoids |
| 80 | α-muurolene | 204.1863 | 227.1756 | -6.4 | 26.63 | [M+Na]^+^ | C_15_H_24_ | 147.11815、 135.11656 | Terpenoids |
| 81 | β-bisabolene | 204.1863 | 227.1756 | -6.4 | 26.63 | [M+Na]^+^ | C_15_H_24_ | 135.11656 、107.08562 | Other categories |
| 82 | β-selinene | 204.1863 | 227.1756 | -6.4 | 26.63 | [M+Na]^+^ | C_15_H_24_ | 147.11815 、135.11656 、107.08562 | Terpenoids |
| 83 | γ-muurolene | 204.1863 | 227.1756 | -6.4 | 26.63 | [M+Na]^+^ | C_15_H_24_ | 147.11815 、135.11656 、107.08562 | Terpenoids |
| 84 | Skimmin | 324.0842 | 347.0734 | -0.9 | 28.56 | [M+Na]^+^ | C_15_H_16_O_8_ | 147.04371 、119.04870 | Coumarins |
| 85 | Bergaptol | 202.0258 | 203.033 | -4.2 | 29.24 | [M+H]^+^ | C_11_H_6_O_4_ | 203.03328 、175.03995、 159.04380、147.04331 、131.04840 | Coumarins |
| 86 | Oxypeucedanin hydrate | 304.0946 | 305.1019 | -0.1 | 29.24 | [M+H]^+^ | C_16_H_16_O_6_ | 305.10232 、203.03328 、175.03995、159.04380 、147.04331 | Coumarins |
| 87 | Xanthotoxol | 202.0258 | 203.033 | -4.2 | 29.24 | [M+H]^+^ | C_11_H_6_O_4_ | 203.03328 、175.03995 、147.04331 | Coumarins |
| 88 | Diosphenol | 168.1167 | 191.1059 | 8.6 | 30.32 | [M+Na]^+^ | C_10_H_16_O_2_ | 609.18005 、463.12224 、301.07040、 257.04358 、153.01755 | Terpenoids |
| 89 | Byakangelicol | 316.0937 | 317.101 | -3.1 | 30.53 | [M+H]^+^ | C_17_H_16_O_6_ | 233.04399、 231.02880 、215.03395、203.03406 | Coumarins |
| 90 | Byakangelicin | 334.1044 | 357.0937 | -2.3 | 30.53 | [M+Na]^+^ | C_17_H_18_O_7_ | 233.04399 、231.02880、 215.14170、203.03406 、187.03859 | Coumarins |
| 91 | Atractylenolide II | 232.1457 | 233.153 | -2.6 | 30.58 | [M+H]^+^ | C_15_H_20_O_2_ | 233.15105 、215.14170 、187.14704、159.11634 、133.10323、 131.08515、 119.08471 、117.06950 、107.08487、105.06949 | Other categories |
| 92 | Bicyclo[6.3.0]undeca-1,7-dien-3-one, 5,5-dimethyl- | 190.1353 | 191.1426 | -2.3 | 30.59 | [M+H]^+^ | C_13_H_18_O | 191.14078 、173.13163、 157.10028、149.09417、 147.11573 、145.10057 | Other categories |
| 93 | DAMASCONE | 192.1532 | 215.1425 | 8.5 | 30.59 | [M+Na]^+^ | C_13_H_20_O | 215.14170、 149.09417 、145.10057、133.10323 、119.08417、 107.08487、 105.06949 | Phenylpropanoids |
| 94 | 7-HYDROXYCOUMARIN | 162.0312 | 163.0385 | -3.1 | 34.47 | [M+H]^+^ | C_9_H_6_O_3_ | 163.03865、 145.02702、 121.02795 | Coumarins |
| 95 | tricin | 330.0709 | 331.0782 | -9.2 | 34.47 | [M+H]^+^ | C_17_H_14_O_7_ | 287.05003、 191.03435 | Flavonoids |
| 96 | 3,5,6-trihydroxy-4',7'-dimethoxyflavone | 330.0709 | 331.0782 | -9.2 | 34.47 | [M+H]^+^ | C_17_H_14_O_7_ | 287.05003 、183.02449 | Flavonoids |
| 97 | 4-HYDROXYCOUMARIN | 162.0312 | 163.0385 | -3.1 | 34.47 | [M+H]^+^ | C_9_H_6_O_3_ | 163.03865 、135.04376、 121.02795 | Coumarins |
| 98 | 5,7-Dimethoxycoumarin | 206.058 | 207.0652 | 0.3 | 34.47 | [M+H]^+^ | C_11_H_10_O_4_ | 207.06491 、177.05453 、163.07523、161.05888、 151.03091 、149.02297、121.02795、107.04867 、105.03337 | Coumarins |
| 99 | Citropten | 206.058 | 207.0652 | 0.3 | 34.47 | [M+H]^+^ | C_11_H_10_O_4_ | 207.06491 、177.05453 、163.07523、151.03091 、149.02297、 121.02795、107.04867 、105.03337 、103.01767 | Coumarins |
| 100 | Iristectorigenin A | 330.0709 | 331.0782 | -9.2 | 34.47 | [M+H]^+^ | C_17_H_14_O_7_ | 287.05003 、183.02449 、149.05896 | Flavonoids |
| 101 | Limetin | 206.058 | 207.0652 | 0.3 | 34.47 | [M+H]^+^ | C_11_H_10_O_4_ | 207.06491 、177.05453 、163.07523、 163.03865 、161.05888 、151.03091、149.02297、 121.02795 、107.04867、105.03337 、103.01767 | Coumarins |
| 102 | α-rotunol | 234.1616 | 235.1689 | -1.6 | 35.96 | [M+H]^+^ | C_15_H_22_O_2_ | 235.16894 、217.15743 、199.14998、 175.11055 、163.11094 、151.11209、149.09358、 133.10096 、107.08514、107.04859 、105.06915 | Terpenoids |
| 103 | sugeonol | 234.1615 | 235.1688 | -1.9 | 37.68 | [M+H]^+^ | C_15_H_22_O_2_ | 235.16692 、217.15785 、207.17529、 205.15875 、201.12639、 199.14807、 185.13157 、135.11699、133.10097、 121.10043 | Other categories |
| 104 | 4,7-dimethyl-1-tetralone | 174.104 | 175.1113 | -2.6 | 37.69 | [M+H]^+^ | C_12_H_14_O | 175.11266 、159.08109、 147.11620、 141.06896 、133.06510 、131.08517、 119.08517 、117.06900 、115.05422、105.06945 | Other categories |
| 105 | cadalene | 198.1403 | 199.1475 | -2.9 | 37.69 | [M+H]^+^ | C_15_H_18_ | 199.14807 、183.11849 、159.11682、157.10096、 155.08521 、145.10062、143.08477 、141.06896 、131.08517、129.06861 、119.08517 、115.05422 | Terpenoids |
| 106 | Geranylacetone | 194.1692 | 217.1584 | 9.9 | 37.69 | [M+Na]^+^ | C_13_H_22_O | 177.16347 、149.13240 、135.11699、133.10097 、123.11675 、121.10043、 19.08517 、109.10081、 107.08538 、105.06945 | Other categories |
| 107 | Nerylacetone | 194.1692 | 217.1584 | 9.9 | 37.69 | [M+Na]^+^ | C_13_H_22_O | 217.15785 、177.16347、 135.11699、121.10043 、119.08517 、109.10081、107/08538 、105.06945 | Other categories |
| 108 | calamenene | 202.1718 | 203.1791 | -1.8 | 37.87 | [M+H]^+^ | C_15_H_22_ | 159.11653 、147.11641 、145.10051、119.08492 、109.10236 、105.06929 | Terpenoids |
| 109 | cypera-2,4-diene | 202.1718 | 203.1791 | -1.8 | 37.87 | [M+H]^+^ | C_15_H_22_ | 201.16343、 173.13221 、147.11641、145.10051、 119.08492、 107.08586 | Other categories |
| 110 | germacrone | 218.1667 | 219.174 | -1.7 | 37.91 | [M+H]^+^ | C_15_H_22_O | 219.17283、 201.16201、 161.13197、159.11642 | Other categories |
| 111 | 5-ISOPROPYL-2-METHYLPHENOL | 150.1044 | 151.1117 | -0.3 | 37.92 | [M+H]^+^ | C_10_H_14_O | 151.11321、 109.06565 | Terpenoids |
| 112 | (S)-2-Methyl-5-(prop-1-en-2-yl)cyclohex-2-enone | 150.1044 | 151.1117 | -0.3 | 37.92 | [M+H]^+^ | C_1_0H_1_4O | 151.11321 、109.06565 | Terpenoids |
| 113 | Thymol | 150.1044 | 151.1117 | -0.3 | 37.92 | [M+H]^+^ | C_10_H_14_O | 151.11321 、109.06565 | Phenols |
| 114 | Oxypeucedanin | 286.0852 | 287.0924 | 3.6 | 39.47 | [M+H]^+^ | C_16_H_14_O_5_ | 203.03299 、147.04499 | Coumarins |
| 115 | Atractylenolide I | 230.1304 | 231.1377 | -1.2 | 39.55 | [M+H]^+^ | C_15_H_18_O_2_ | 231.13763 、215.10467 、175.07400、 161.05963 、133.10118 、119.08515、117.06987、 109.10136 、107.08499、105.06970 、103.05404 | Other categories |
| 116 | Dehydrocostus lactone | 230.1304 | 231.1377 | -1.2 | 39.55 | [M+H]^+^ | C_15_H_18_O_2_ | 215.10467 、203.10594 、175.07400、 173.09737 、107.08499 、105.06970、103.05404 | Other categories |
| 117 | 2,4,4-trimethyl-3-(3-oxobutyl)cyclohex-2-en-1-one | 208.1485 | 231.1377 | 9.2 | 39.55 | [M+Na]^+^ | C_13_H_20_O_2_ | 231.13763 、133.10118 、105.06970 | Terpenoids |
| 118 | Camphor | 152.1216 | 175.1108 | 8.5 | 39.78 | [M+Na]^+^ | C_10_H_16_O | 137.09622 、123.08012 、119.08494 | Terpenoids |
| 119 | 2-(4-methylphenyl)propan-2-ol | 150.1059 | 173.0952 | 8.5 | 40.69 | [M+Na]^+^ | C_10_H_14_O | 119.08569 、117.06899 | Other categories |
| 120 | sugeonyl acetate | 276.172 | 277.1792 | -2.1 | 41.49 | [M+H]^+^ | C_17_H_24_O_3_ | 233.15499 、217.15770 、215.14217、 199.14829 、189.12629 、171.11629、 161.13243、 161.09640、145.10078 、133.10037 | Other categories |
| 121 | Limonin | 470.1937 | 471.201 | -0.8 | 41.62 | [M+H]^+^ | C_26_H_30_O_8_ | 471.20301 、453.19579 、425.19592、 407.18458 | Terpenoids |
| 122 | isopatchoula-3,5-diene | 202.1718 | 203.1791 | -1.5 | 42.25 | [M+H]^+^ | C_15_H_22_ | 203.17875 、201.16481、 175.14773、161.13357 、147.11667 、119.08532 | Other categories |
| 123 | isocurcumenol | 234.1618 | 235.1691 | -0.7 | 42.83 | [M+H]^+^ | C_15_H_22_O_2_ | 235.16734 、217.15782 、147.08181、119.08524 | Other categories |
| 124 | Neral | 152.1216 | 175.1108 | 8.6 | 42.87 | [M+Na]^+^ | C_10_H_16_O | 107.08569 、105.06947 | Other categories |
| 125 | (-)-trans-Carveol | 152.1216 | 175.1108 | 8.6 | 42.87 | [M+Na]^+^ | C_10_H_16_O | 119.08524 、107.08569 | Terpenoids |
| 126 | CARVEOL | 152.1216 | 175.1108 | 8.6 | 42.87 | [M+Na]^+^ | C_10_H_16_O | 119.08524 、107.08569 | Terpenoids |
| 127 | cis-2,8-Menthadien-1-ol | 152.1216 | 175.1108 | 8.6 | 42.87 | [M+Na]^+^ | C_10_H_16_O | 107.08569 、105.06947 | Terpenoids |
| 128 | α-Cyclocitral | 152.1216 | 175.1108 | 8.6 | 42.87 | [M+Na]^+^ | C_10_H_16_O | 119.08524 、107.08569 、105.06947 | Other categories |
| 129 | cyperolone | 236.1767 | 237.1839 | -4.1 | 43.57 | [M+H]^+^ | C_15_H_24_O_2_ | 205.15883 、177.12801 、175.11181、173.13254 、161.13237 、151.11041、137.09646 、133.10117 | Terpenoids |
| 130 | Tocopherol | 490.2201 | 491.2274 | -9.9 | 43.59 | [M+H]^+^ | C_24_H_34_N_4_O_5_S | 223.14850 、213.12710 、201.12695、 173.13254 、173.09532 、171.11606、161.09591、 149.13232 、145.10087、143.08463 、141.06985、 135.11600 133.10117 、131.08500 、129.06875、 121.10026 、117.06949 、115.05391、 107.08498、 105.06937 、103.05374 | Phenols |
| 131 | (+)－Nootkatone | 218.1663 | 219.1735 | -3.7 | 43.59 | [M+H]^+^ | C_15_H_22_O | 217.15819 、203.14393 、177.12801、 175.11181 、173.13254 、163.11178、157.10088、 151.11041 、147.11590、 145.10087、 137.09646、 135.08006 131.08500、 107.08498 | Terpenoids |
| 132 | mustakone | 218.1663 | 219.1735 | -3.7 | 43.59 | [M+H]^+^ | C_15_H_22_O | 217.15819、 203.14393 、177.12801、175.11181 、149.13232、 147.11590、 145.10087 、107.08498 、105.06937 | Other categories |
| 133 | patchoulenone | 218.1663 | 219.1735 | -3.7 | 43.59 | [M+H]^+^ | C_15_H_22_O | 217.15819 、203.14393 、189.16359、187.14712 、173.13254 、171.11606、163.11178 、161.09591 、135.11600、133.10117 、121.10026 、107.08498、 105.06937 | Terpenoids |
| 134 | rotundone | 218.1663 | 219.1735 | -3.7 | 43.59 | [M+H]^+^ | C_15_H_22_O | 217.15819、 203.14393 、189.16359、177.12801 、173.13254 、163.11178、161.09591 、157.10088 、151.11041、149.13232 、147.11590 、145.10087 137.09646 、135.08006 、133.06440、131.08500 、121.10026 、117.06949、109.06382 、107.08498 、105.06937 | Other categories |
| 135 | Glabrone | 336.1012 | 337.1085 | 4.2 | 43.67 | [M+H]^+^ | C_20_H_16_O_5_ | 337.10947、 189.09274 | Flavonoids |
| 136 | 4-Ethyl-o-xylene | 134.109 | 135.1163 | -3.7 | 44.52 | [M+H]^+^ | C_10_H_14_ | 133.10065 、119.08531、 109.10063、107.08494 、105.06959 | Other categories |
| 137 | aristolone | 218.1667 | 219.174 | -1.7 | 44.52 | [M+H]^+^ | C_15_H_22_O | 161.13197、 149.13281 、135.11616、123.11624 、121.10109、 109.10063、107.08494 | Other categories |
| 138 | O-CYMENE | 134.109 | 135.1163 | -3.7 | 44.52 | [M+H]^+^ | C_10_H_14_ | 135.11616 、119.08531 、107.08494、105.06959 | Other categories |
| 139 | P-CYMENE | 134.109 | 135.1163 | -3.7 | 44.52 | [M+H]^+^ | C_10_H_14_ | 135.11712 、133.10065 、119.08531、 | Other categories |
| 140 | 10,12-peroxycalamenene | 232.1458 | 233.1531 | -2.3 | 44.76 | [M+H]^+^ | C_15_H_20_O_2_ | 233.15289 、191.10773、 | Other categories |
| 141 | Eugenol | 164.0854 | 187.0746 | 8.9 | 44.85 | [M+Na]^+^ | C_10_H_12_O_2_ | 133.06446 、121.06481 、115.05372、 105.05952 | Phenylpropanoids |
| 142 | isoeugenol | 164.0854 | 187.0746 | 8.9 | 44.85 | [M+Na]^+^ | C_10_H_12_O_2_ | 137.05911 、133.06446 、109.06407、 105.06952 | Phenylpropanoids |
| 143 | Obacunone | 454.1986 | 455.2059 | -1.1 | 44.85 | [M+H]^+^ | C_26_H_30_O_7_ | 455.20487 、437.19459 、411.21500、 409.20048、 177.09089 、163.07359、 133.06446、 105.06952 | Terpenoids |
| 144 | Phenethyl acetate | 164.0854 | 187.0746 | 8.9 | 44.85 | [M+Na]^+^ | C_10_H_12_O_2_ | 187.07439 、105.06952 、103.05417 | Other categories |
| 145 | caryophyllene-α-oxide | 220.1812 | 221.1884 | -7 | 45.97 | [M+H]^+^ | C_15_H_24_O | 147.11641 、145.10051 | Terpenoids |
| 146 | cyperol | 220.1812 | 221.1884 | -7 | 45.97 | [M+H]^+^ | C_15_H_24_O | 159.11653 、147.11641、 145.10051、 121.10053 、119.08492 、105.06929 | Terpenoids |
| 147 | isocyperol | 220.1812 | 221.1884 | -7 | 45.97 | [M+H]^+^ | C_15_H_24_O | 159.11653 、147.11641、145,.10051、121.10053 、119.08492 、105.06929 | Terpenoids |
| 148 | Spathulenol | 220.1812 | 221.1884 | -7 | 45.97 | [M+H]^+^ | C_15_H_24_O | 201.16343 、147.11641 、121.10053、119.08492 、105.06929 | Other categories |
| 149 | METHYL EUGENOL | 178.0988 | 179.1061 | -3.1 | 51.24 | [M+H]^+^ | C_11_H_14_O_2_ | 179.10626、 163.07551 、107.07924 | Phenylpropanoids |
| 150 | Farnesylacetone | 262.2292 | 263.2365 | -1.8 | 52.89 | [M+H]^+^ | C_18_H_30_O | 14909604 、133.10094 、109.06413 、105.06907 | Other categories |
| 151 | TERPINEN-4-OL | 154.1372 | 177.1265 | 8.4 | 53.19 | [M+Na]^+^ | C_10_H_18_O | 177.12580 、111.08078 | Terpenoids |
| 152 | oleic acid | 282.2563 | 283.2636 | 1.6 | 53.2 | [M+H]^+^ | C_18_H_34_O_2_ | 207.03277、 152.06116 、141.06951 | Other categories |
| 153 | Ostruthine | 298.1567 | 299.164 | -0.6 | 53.2 | [M+H]^+^ | C_19_H_22_O_3_ | 243.10564、 213.05313、 105.06952 | Phenylpropanoids |
|  |  |  |  |  |  |  |  |  |  |
